# Association between endothelin-1 and systemic lupus erythematosus: insights from a case–control study

**DOI:** 10.1038/s41598-023-43350-0

**Published:** 2023-09-25

**Authors:** Rong Li, Chan Yang, Yang-Yang Tang, Da-Cheng Wang, Wang-Dong Xu, An-Fang Huang

**Affiliations:** 1 Department of Evidence-Based Medicine, School of Public Health, Southwest Medical University, 1 Xianglin Road, Luzhou, 646000 Sichuan China; 2 Department of Rheumatology and Immunology, The Affiliated Hospital, Southwest Medical University, 25 Taiping Road, Luzhou, 646000 Sichuan China

**Keywords:** Genetics, Immunology, Biomarkers, Rheumatology

## Abstract

Systemic lupus erythematosus (SLE) is a chronic rheumatic disorder. Endothelin-1, a vasoconstrictor, belongs to the endothelin family and is associated with vascular-related damages. To date, association between ET-1 and pathogenesis of SLE remains unclear. This case–control study was carried out by 314 SLE, 252 non-SLE diseases patients and 500 healthy controls. Serum ET-1, CCN3, IL-28B levels were detected by ELISA, and *ET-1* gene polymorphisms (rs5369, rs5370, rs1476046, rs2070699, rs2071942, rs2071943, rs3087459, rs4145451, rs6458155, rs9369217) were genotyped with Kompetitive Allele-Specific PCR. SLE patients had high levels of ET-1, which were correlated with some clinical, laboratory features. Serum CCN3, IL-28B levels were higher in SLE patients, and ET-1 levels were positively correlated with the two cytokines. Rs5370, rs1476046, rs2070699, rs2071942, rs2071943, rs3087459, rs6458155 and rs2070699 were associated with SLE risk. Rs2070699 (T, TT) was related to SLE patients with alopecia. Rs5370 (T, TT, TG), rs1476046 (G,GA), rs2071942 (G,GA) and rs2071943 (G,GA) were associated with SLE patients with pericarditis, pyuria and fever manifestation, respectively. Rs3087459 (CC) and rs9369217 (TC) were related to SLE patients with positive anti-SSB antibody. Rs5369 (AA) was associated with IgG and CRP levels in SLE patients. In conclusion, elevated serum ET-1 in SLE patients may be a potential disease marker, and its gene polymorphisms were related to SLE susceptibility.

## Introduction

Systemic lupus erythematosus (SLE) is an autoimmune disease characterized by dysregulated production of autoantibodies and inflammatory cytokines^1^. Clinical manifestations caused by systemic inflammation and tissue damage in SLE are complex and different, for instance, damages in skin, kidney, joint, blood vessel^2^. Clear pathogenesis of SLE remains obscure, but genetic susceptibility, environmental factors and sex hormones have been demonstrated to play pivotal roles in. To date, genome-wide association studies (GWASs) identified about 180 genetic loci associated with SLE susceptibility^3^.

Endothelin-1 (ET-1), the main component of endothelin family, consists of 21 amino acids and two intramolecular disulphide bonds. ET-1 is expressed in vascular endothelial cells, smooth muscle cells, cardiomyocytes, fibroblasts and macrophages^4^. Being an endogenous long-acting vasoconstrictor, ET-1 promotes growth factors production and stimulates proliferation and contraction of vascular endothelial cells, smooth muscle cells and fibroblasts^5^. Interestingly, SLE is associated with vascular damages resulted from endothelial dysfunction, such as vasculitis and Raynaud’s phenomenon. ET-1 derived from endothelial cells may lead to endothelial dysfunction^6^. In rheumatoid arthritis (RA) patients, higher serum ET-1 levels were detected when compared to controls, and were positively related to C-reactive protein (CRP) levels^7^. Levels of ET-1 were increased in both psoriasis vulgaris and systemic sclerosis (SSc) patients^8,9^. These findings indicated that ET-1 is excessively expressed in inflammatory autoimmune diseases. To date, there is limited discussion regarding ET-1 and SLE, and it is still unclear whether ET-1 gene polymorphisms are associated with increased risk of SLE.

Nephroblastoma overexpressed (NOV, CCN3) is highly expressed in some immune cells, such as regulatory T cells^10^. CCN3 is capable of inducing angiogenesis by binding ligands αvβ3 and α5β1, and thereby promoting adhesion and migration of endothelial cells^11^. Elevated levels of CCN3 were reported in RA, SSc patients^12^. Type I interferon is associated with development of SLE^13^. When expression of type I interferon and its receptors are inhibited in lupus mice, autoantibodies production and disease development are suppressed^13^. Type III interferons, including IL-28A, IL-28B, IL-29, are functionally similar to type I interferon. IL-28B involves in antiviral and antitumor responses, and contributes to development of immune-related disorders^14^. For example, levels of IL-28B were increased in SLE patients and were related to disease activity^15^. However, the relationship between ET-1 and CCN3, as well as the relationship between ET-1 and IL-28B, in SLE remains obscure.

Therefore, role of ET-1 in SLE is not clear, and whether ET-1 regulates CCN3, IL-28B, and then contributes to SLE pathogenesis is not discussed. In this study, we conducted a case–control study with SLE patients and healthy controls to reveal serum levels of ET-1 in SLE, gene polymorphisms of ET-1 in SLE. Second, we examined serum levels of CCN3, IL-28B in SLE patients, and discussed association of ET-1 and CCN3, IL-28B. If the two inflammatory components (CCN3, IL-28B) were abnormally expressed in SLE patients and there is significant association of ET-1 and CCN3, ET-1 and IL-28B, in the future, we will conduct functional study to clarify how ET-1 contributes to SLE development, and whether ET-1 contributes to SLE development by regulating CCB3, IL-28B.

## Results

### ET-1 serum levels in SLE from training cohort

**Figure 1:**
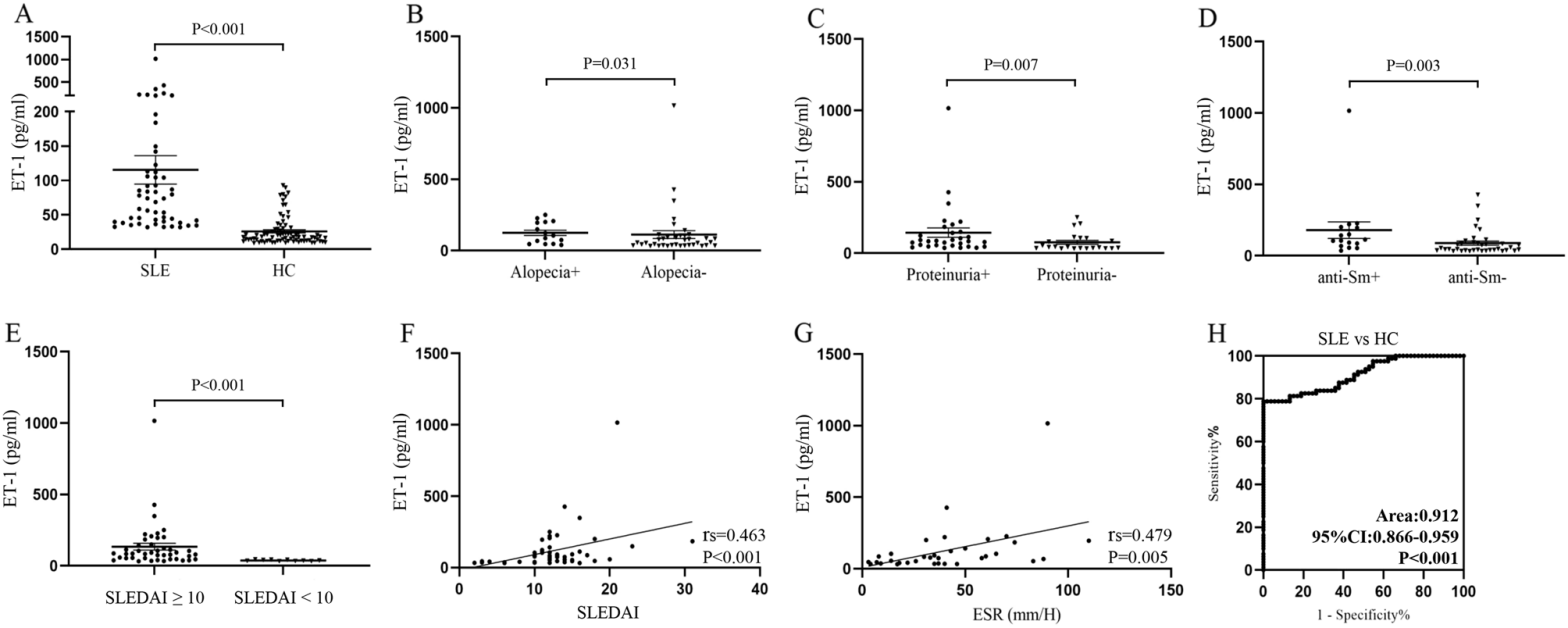
Comparison of ET-1 levels between SLE patients and healthy controls in the training cohort. (**A**) Serum ET-1 levels in 53 SLE patients and 80 healthy individuals were examined by ELISA. Each symbol stands for an independent sample. (**B**–**D**) ET-1 levels in SLE patients distributed according to alopecia, proteinuria and anti-Sm. (**E**) Difference of serum levels of ET-1 in SLE patients with less active disease and active disease. (**F**–**G**) Relationship between ET-1 levels and SLEDAI, ESR levels. (**H**) Receiver operating characteristic curve analysis of serum ET-1 as a biomarker for SLE.

To study association between serum levels of ET-1 and SLE, a training cohort with 53 SLE patients and 80 healthy controls was analyzed. Clinical and laboratory characteristics of all subjects are shown in Table 1. Age of SLE patients and controls was 38.00 (27.00–49.00) years and 37.00 (34.00–40.00) years, and there was no difference in age of the two groups (Z = − 0.145, *P* = 0.884). Moreover, gender of SLE and healthy controls was matched (*χ*^2^ = 2.847, *P* = 0.092). ET-1 serum levels were significantly higher in SLE patients compared to healthy controls (74.11 (40.00–117.82) vs 17.35 (12.18–29.28) pg/ml, Z = − 8.029, *P* < 0.001, Fig. 1A). Patients with alopecia (N = 15) had increased serum levels of ET-1 when compared with that in patients without alopecia (N = 38) (*P* = 0.031, Fig. 1B). Patients with proteinuria (N = 31) had increased serum levels of ET-1 when compared with that in patients without proteinuria (N = 22) (*P* = 0.007, Fig. 1C). Similarly, SLE patients with positive anti-Sm antibody (N = 16) revealed elevated levels of ET-1 in serum than that in patients with negative anti-Sm antibody (N = 37) (*P* = 0.003, Fig. 1D). Moreover, SLE patients with active disease (SLEDIA ≥ 10) had higher ET-1 levels compared to patients with less active disease (SLEDIA < 10) (*P* < 0.001, Fig. 1E). Correlation analysis revealed positive correlation between serum ET-1 levels and SLEDAI score (r_s_ = 0.463, *P* < 0.001, Fig. 1F), ESR levels (r_s_ = 0.479, *P* = 0.005, Fig. 1G). Other clinical and laboratory features in SLE patients are not associated with serum ET-1 levels (Supplementary table 1 and 2). To evaluate ability of serum ET-1 to distinguish SLE patients from healthy individuals, we performed ROC curve analysis, which showed area under ROC curve (AUC) 0.912 (95% CI = 0.866–0.959) (*P* < 0.001, Fig. 1H).

**Table 1:** Characteristics of SLE patients and control groups.

Characteristics	SLE	RA	OA	SS	AS	SSc	HC
Number (n)	314	90	95	55	38	17	500
Age (years)	38.00 (27.00–49.00)	56.00 (50.00–66.00)	57.00 (50.00–67.00)	54.00 (46.25–60.75)	32.00 (25.00–45.00)	46.00 (43.00–51.50)	37.00 (34.00–40.00)
Female (%)/male (%)	89.17/10.83	70.21/29.79	83.87/16.13	90.90/9.09	21.21/78.79	40.00/60.00	92.60/7.40
Lupus headache, n (%)	21 (6.6)	–	–	–	–	–	–
Vasculitis, n (%)	28 (8.92)	–	–	–	–	–	–
Arthritis, n (%)	158 (50.32)	–	–	–	–	–	–
Myositis, n (%)	31 (9.87)	–	–	–	–	–	–
Rash, n (%)	132 (42.04)	–	–	–	–	–	–
Alopecia, n (%)	98 (31.21)	–	–	–	–	–	–
Oral ulcer, n (%)	45 (14.33)	–	–	–	–	–	–
Pleurisy, n (%)	25 (7.9)	–	–	–	–	–	–
Pericarditis, n (%)	25 (7.9)	–	–	–	–	–	–
Fever, n (%)	61 (19.43)	–	–	–	–	–	–
Hypocomplementemia, n (%)	157 (50.00)	–	–	–	–	–	–
ds-DNA + , n (%)	70 (22.29)	–	–	–	–	–	–
Thrombocytopenia, n (%)	47 (14.99)	–	–	–	–	–	–
Leukopenia, n (%), n (%)	36 (11.46)	–	–	–	–	–	–
Hematuria, n (%)	107 (34.08)	–	–	–	–	–	–
Proteinuria, n (%)	154 (49.04)	–	–	–	–	–	–
Pyuria, n (%)	27 (8.5)	–	–	–	–	–	–
C3 (g/L)	0.73 (0.46–0.94)	–	–	–	–	–	–
C4 (g/L)	0.14 (0.07–0.22)	–	–	–	–	–	–
ESR (mm/H)	25.00 (11.00–51.00)	–	–	–	–	–	–
RF (IU/ml)	9.70 (8.40–12.30)	–	–	–	–	–	–
IgA (mg/L)	2.55 (1.82–3.33)	–	–	–	–	–	–
IgM (mg/L)	1.01 (0.69–1.44)	–	–	–	–	–	–
IgG (g/L)	14.24 (10.58–19.75)	–	–	–	–	–	–
CRP (mg/L)	3.00 (0.5–14.59)	–	–	–	–	–	–

SLE, systemic lupus erythematosus; RA, rheumatoid arthritis; OA, osteoarthritis; SS, Sjögren’s syndrome; AS, ankylosing spondylitis; SSc, systemic sclerosis; HC, healthy controls; ESR, erythrocyte sedimentation rate; RF, rheumatoid factor; CRP, C-reactive protein.

### Elevated serum levels of ET-1 in SLE from validation cohort

**Figure 2:**
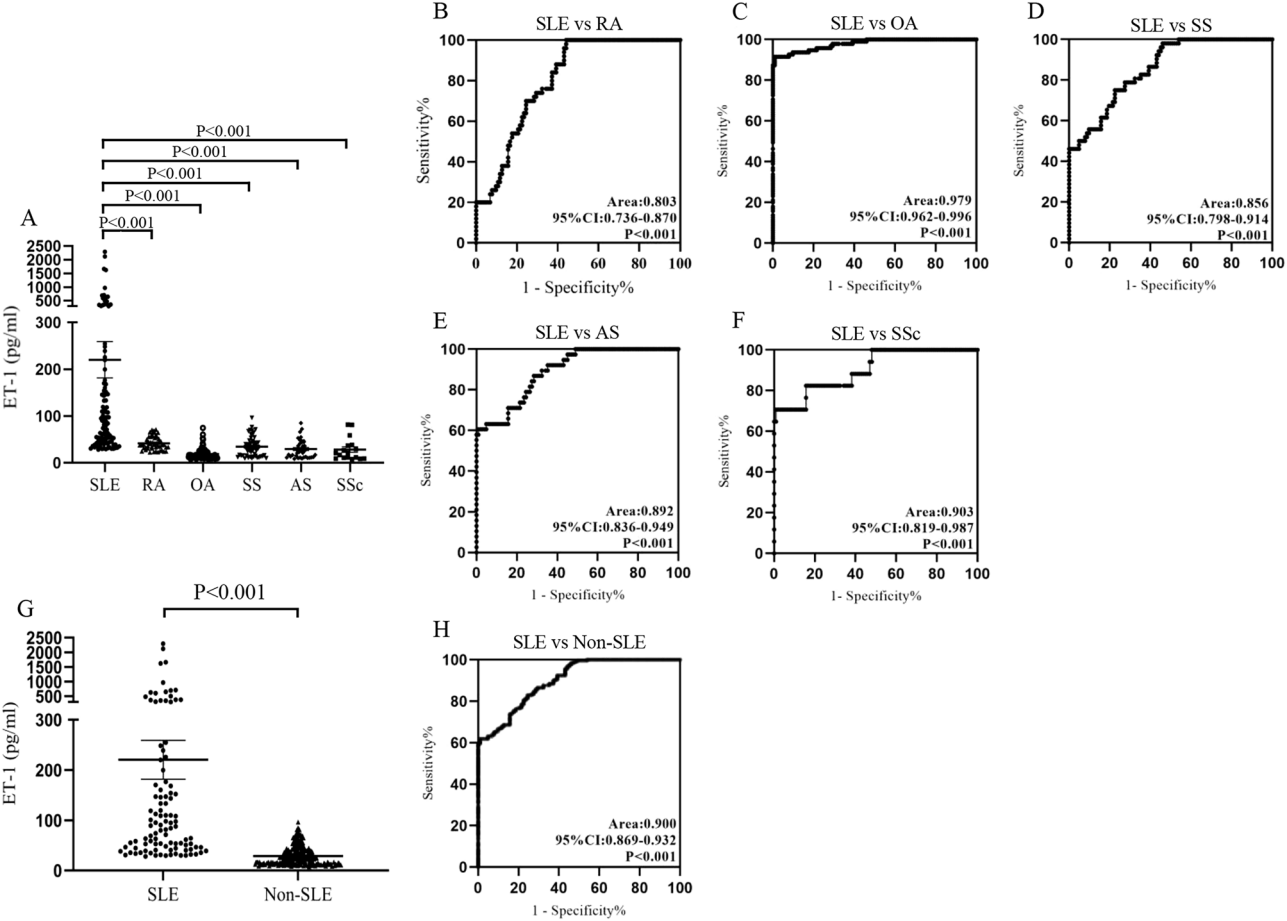
ET-1 levels in SLE patients from validation cohort. (**A**) Comparison of serum ET-1 levels in SLE patients (N = 102) and diseases controls (N = 252, including 90 RA, 95 OA, 55 SS, 38 AS, 17 SSc) by ELISA. (**B**–**F**) Receiver operating characteristic curve analysis was used to assess potential of serum ET-1 in differentiating SLE from RA, OA, SS, AS, and SSc. (**G**) Analysis of difference in serum ET-1 between SLE and non-SLE patients. (**H**) Receiver operating characteristic curve analysis of serum ET-1 between SLE and non-SLE patients.

To evaluate potential of serum ET-1 as a biomarker for SLE, a validation cohort including 102 SLE patients and 252 non-SLE diseases patients was discussed further (90 RA, 95 osteoarthritis (OA), 55 sjogren syndrome (SS), 38 anlylosing spondylitis (AS), 17 SSc). Information about age and sex of these diseases patients is shown in Table 1. Results indicated that serum ET-1 levels in SLE patients were significantly higher than that in other rheumatic diseases (all *P* < 0.001, Fig. 2A). AUC was 0.803 by ROC curve analysis when comparing serum levels of ET-1 between SLE with RA patients (Fig. 2B). Similarly, serum ET-1 in SLE patients compared to that in OA, SS, AS, SSc, showed AUC 0.979, 0.856, 0.892 and 0.903, respectively (Fig. 2C–F). We compared serum ET-1 levels between SLE patients and non-SLE patients, showing that there was a significant difference (*P* < 0.001, Fig. 2G) and AUC was 0.900 (95% CI = 0.869–0.932) (Fig. 2H).

### Connection between ET-1 and CCN3, IL-28B levels in SLE

**Figure 3:**
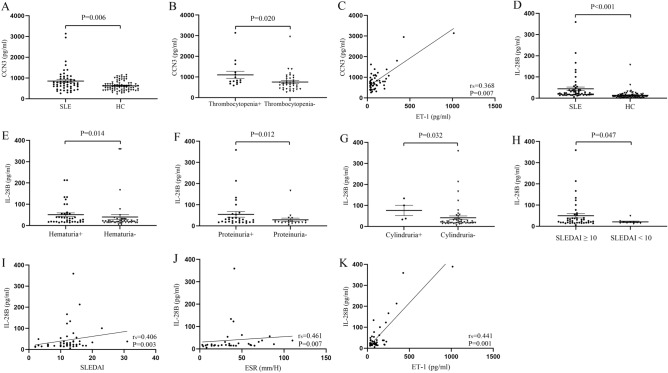
CCN3 and IL-28B levels in SLE patients and healthy controls. (**A**) Serum CCN3 levels were tested by ELISA in 53 SLE patients and 80 healthy controls. (**B**) Serum ET-1 in SLE patients distributed in accordance with thrombocytopenia. (**C**) Correlation between ET-1 and CCN3 levels in SLE patients. (**D**) Serum IL-28B levels were detected by ELISA in 53 SLE patients and 80 healthy controls. (**E**–**G**) Distribution of serum IL-28B in SLE patients with hematuria, proteinuria and cylindruria. (**H**) Difference of serum IL-28B in SLE patients with active disease and less active disease. (**I**–**K**) Relationship between IL-28B levels and SLEDAI score, ESR levels. (H) Correlation between ET-1 and IL-28B levels in SLE patients.

There were increased serum CCN3 levels in SLE patients compared with that in healthy controls (733.67 (553.57–988.08) vs 587.94 (430.06–748.02) pg/ml, Z = − 2.769, *P* = 0.006, Fig. 3A). Lupus patients with thrombocytopenia had higher levels of CCN3 (*P* = 0.020, Fig. 3B) (Supplementary table 3). SLEDAI score and other clinical characteristics in SLE patients were not significantly correlated with CCN3 levels (Supplementary table 4). Correlation between serum levels of ET-1 and CCN3 showed significant correlation (r_s_ = 0.338, *P* = 0.007, Fig. 3C). For IL-28B, its levels were elevated in serum of SLE patients as compared to that in healthy controls (23.30 (16.93–39.12) vs 9.80 (5.34–15.92) pg/ml, Z = -6.974, *P* < 0.001, Fig. 3D). Lupus patients with hematuria, proteinuria, cylindruria had higher levels of IL-28B (*P* = 0.014, Fig. 3E; *P* = 0.012, Fig. 3F; *P* = 0.032, Fig. 3G) (Supplementary table 5). Higher serum IL-28B levels in SLE patients with active disease (SLEDIA ≥ 10) were obtained when compared to patients with less active disease (SLEDIA < 10) (*P* = 0.047, Fig. 3H). Correlation analysis reported that SLEDAI score, ESR levels were positively correlated with IL-28B levels, respectively (r_s_ = 0.406, *P* = 0.003, Fig. 3I; r_s_ = 0.461, *P* = 0.007, Fig. 3J) (Supplementary table 6). ET-1 levels were positively correlated with IL-28B levels in SLE patients (r_s_ = 0.441, *P* = 0.001, Fig. 3K).

### Association of *ET-1* gene single nucleotide polymorphisms (SNPs) with SLE

To discuss whether polymorphisms of *ET-1* gene correlate with SLE risk, a total of 314 lupus patients and 500 age, sex-matched healthy volunteers were selected. Hardy–Weinberg equilibrium (HWE) test showed no deviation in SLE patients and controls for all 10 polymorphisms (*P* > 0.05, Table 2). Information about alleles and genotypes of the SNPs was shown in Table 3. Frequency of rs5370 genotype TG was higher in SLE patients compared to healthy controls (TG vs. GG: OR = 1.443, 95% CI 1.072–1.943, *P* = 0.016). In the dominant model (TT + TG vs GG), frequency of genotypes TT + TG of rs5370 was increased in SLE patients (OR = 1.334, 95% CI 1.005–1.770, *P* = 0.046). For rs1476046, frequency of genotype GG in SLE patients was markedly lower than that in controls [GG vs GA + AA (recessive model): OR = 0.693, 95% CI 0.521–0.920, *P* = 0.011]. Compared with healthy controls, frequency of rs2070699 allele T was declined in SLE patients (T vs G: OR = 0.811, 95% CI 0.664–0.990, P = 0.039). Similarly, decreased frequencies of TT, TG, TT + TG genotypes of rs2070699 were found in SLE patients [TT vs GG: OR = 0.669, 95% CI 0.452–0.986, *P* = 0.042; TG vs GG: OR = 0.674, 95% CI 0.475–0.957, *P* = 0.027; TT + TG vs GG (dominant model): OR = 0.672, 95% CI 0.485–0.931, *P* = 0.017]. Frequency of GG genotype for rs2071942 was increased in SLE patients when compared with that in healthy subjects (GG vs GA: OR = 1.705, 95% CI 1.033–2.812, *P* = 0.037). With respect to rs2071943, there was decreased frequency of GG in SLE patients than that in control group [GG vs. GA + AA (recessive model): OR = 0.707, 95% CI 0.532–0.938, *P* = 0.016]. In the dominant model, higher frequencies of CA + CC genotypes of rs3087459 were found in patients with SLE (CA + CC vs AA: OR = 1.352, 95% CI 1.013–1.805, *P* = 0.040). In addition, SLE patients with rs6458155 had an enhanced frequency of TC genotype (TC vs CC: OR = 1.392, 95% CI 1.021–1.899, *P* = 0.0037). For rs5369, rs4145451, rs9369217 polymorphisms, we discussed the allele and genotypes distribution between SLE cases and healthy controls, showing no significant differences.

**Table 2:** The Hardy–Weinberg’s expectation test in SLE patients and healthy controls for SNPs.

SNPs	SLE		HC	
rs5369	χ^2^ = 4.594	*P* = 0.101	χ^2^ = 4.735	*P* = 0.094
rs5370	χ^2^ = 0.918	*P* = 0.632	χ^2^ = 2.062	*P* = 0.357
rs1476046	χ^2^ = 0.516	*P* = 0.772	χ^2^ = 1.568	*P* = 0.457
rs2070699	χ^2^ = 1.847	*P* = 0.397	χ^2^ = 0.036	*P* = 0.982
rs2071942	χ^2^ = 0.190	*P* = 0.909	χ^2^ = 5.628	*P* = 0.060
rs2071943	χ^2^ = 0.308	*P* = 0.857	χ^2^ = 2.067	*P* = 0.356
rs3087459	χ^2^ = 3.917	*P* = 0.141	χ^2^ = 3.179	*P* = 0.204
rs4145451	χ^2^ = 0.657	*P* = 0.720	χ^2^ = 2.351	*P* = 0.309
rs6458155	χ^2^ = 0.540	*P* = 0.763	χ^2^ = 1.582	*P* = 0.453
rs9369217	χ^2^ = 1.141	*P* = 0.565	χ^2^ = 1.095	*P* = 0.578

SNP, single nucleotide polymorphisms. SLE, systemic lupus erythematosustis; HC, healthy controls.

**Table 3:** Frequencies of alleles and genotypes for ET-1 gene polymorphisms in SLE patients and healthy controls.

Polymorphism			SLE, n (%)	Controls, n (%)	OR (95% Cl)	*P* value
rs5369	Genotype	GG	292 (93.0)	455 (91)	0.770 (0.233–2.546)	0.668
		GA	17 (5.4)	39 (7.8)	0.523 (0.140–1.951)	0.335
		AA	5 (1.6)	6 (1.2)	Reference	
	Allele	G	601 (95.7)	949 (94.9)	1.196 (0.742–1.928)	0.462
		A	27 (4.3)	51 (5.1)	Reference	
	Recessive model	GG	292 (93.0)	455 (91.0)	1.313 (0.772–2.232)	0.315
		GA + AA	22 (7.0)	45 (9.0)	Reference	
	Dominant model	GA + GG	309 (98.4)	494 (98.8)	0.751 (0.227–2.480)	0.638
		AA	5 (1.6)	6 (1.2)	Reference	
rs5370	Genotype	TT	24 (7.7)	48 (9.6)	0.918 (0.541–1.560)	0.753
		TG	143 (45.5)	182 (36.4)	1.443 (1.072–1.943)	0.016
		GG	147 (46.8)	270 (54.0)	Reference	
	Allele	T	191 (30.4)	278 (27.8)	1.135 (0.912–1.413)	0.257
		G	437 (69.6)	722 (72.2)	Reference	
	Recessive model	TT	24 (7.7)	48 (9.6)	0.779 (0.497–1.300)	0.340
		TG + GG	290 (92.3)	452 (90.4)	Reference	
	Dominant model	TT + TG	167 (53.2)	230 (46.0)	1.334 (1.005–1.770)	0.046
		GG	147 (46.8)	270 (54.0)	Reference	
rs1476046	Genotype	GG	134 (42.7)	259 (51.8)	0.825 (0.506–1.344)	0.439
		GA	148 (47.1)	190 (38.0)	1.241 (0.759–2.029)	0.388
		AA	32 (10.2)	51 (10.2)	Reference	
	Allele	G	416 (66.2)	708 (70.8)	0.823 (0.665–1.019)	0.074
		A	212 (33.8)	292 (29.2)	Reference	
	Recessive model	GG	134 (42.7)	259 (51.8)	0.693 (0.521–0.920)	0.011
		GA + AA	180 (57.3)	241 (48.2)	Reference	
	Dominant model	GA + GG	282 (89.8)	449 (89.8)	1.001 (0.628–1.596)	0.997
		AA	32 (10.2)	51 (10.2)	Reference	
rs2070699	Genotype	TT	85 (27.1)	150 (30.0)	0.669 (0.454–0.986)	0.042
		TG	140 (44.6)	245 (49.0)	0.674 (0.475–0.957)	0.027
		GG	89 (28.3)	105 (21.0)	Reference	
	Allele	T	310(49.4)	546 (54.6)	0.811 (0.664–0.990)	0.039
		G	318 (50.6)	454 (45.4)	Reference	
	Recessive model	TT	85 (27.1)	150 (30.0)	0.866 (0.633–1.185)	0.369
		TG + GG	229 (72.9)	350 (70.0)	Reference	
	Dominant model	TT + TG	225 (71.7)	395 (79.0)	0.672 (0.485–0.931)	0.017
		GG	89 (28.3)	105 (21.0)	Reference	
rs2071942	Genotype	GG	146 (46.5)	264 (52.8)	1.185 (0.725–1.938)	0.499
		GA	140 (44.6)	176 (35.2)	1.705 (1.033–2.812)	0.037
		AA	28 (8.9)	60 (12.0)	Reference	
	Allele	G	432 (68.8)	704 (70.4)	0.927 (0.746–1.151)	0.491
		A	196(31.2)	296 (29.6)	Reference	
rs2071943	Recessive model	GG	146 (46.5)	264 (52.8)	0.777 (0.585–1.031)	0.080
		GA + AA	168 (53.5)	236 (47.2)	Reference	
	Dominant model	GA + GG	286 (91.1)	440 (88.0)	1.393 (0.868–2.234)	0.169
		AA	28 (8.9)	60 (12.0)	Reference	
	Genotype	GG	143 (45.6)	271 (54.2)	0.873 (0.528–1.445)	0.598
		GA	142 (45.2)	181 (36.2)	1.299 (0.799–2.164)	0.316
		AA	29 (9.2)	48 (9.6)	Reference	
rs3087459	Allele	G	428 (68.2)	723 (72.3)	0.820 (0.660–1.019)	0.074
		A	200 (31.8)	277 (27.7)	Reference	
	Recessive model	GG	143 (45.6)	271 (54.2)	0.707 (0.532–0.938)	0.016
		GA + AA	171 (54.4)	229 (45.8)	Reference	
	Dominant model	GA + GG	285 (90.8)	452 (90.4)	1.044 (0.643–1.694)	0.863
		AA	29 (9.2)	48 (9.6)	Reference	
	Genotype	CC	8 (2.6)	10 (2.0)	1.435 (0.556–3.700)	0.455
		CA	127 (40.4)	169 (33.8)	1.348 (1.004–1.808)	0.047
		AA	179 (57.0)	321 (64.2)	Reference	
rs4145451	Allele	C	143 (22.8)	189(18.9)	1.265 (0.991–1.616)	0.059
		A	485 (77.2)	811 (81.1)	Reference	
	Recessive model	CC	8 (2.6)	10(2)	1.281 (0.500–3.281)	0.606
		CA + AA	306 (97.4)	490 (98.0)	Reference	
	Dominant model	CA + CC	135 (43.0)	179 (35.8)	1.352 (1.013–1.805)	0.040
		AA	179 (57.0)	321 (64.2)	Reference	
	Genotype	CC	93 (29.6)	163 (32.6)	1.161 (0.772–1.748)	0.473
		CA	165 (52.6)	223 (44.6)	1.506 (1.032–2.198)	0.034
		AA	56 (17.8)	114(22.8)	Reference	
rs6458155	Allele	C	351 (55.9)	549 (54.9)	1.041 (0.852–1.272)	0.695
		A	277 (44.1)	451 (45.1)	Reference	
	Recessive model	CC	93 (29.6)	163 (32.6)	0.870 (0.641–1.182)	0.373
		CA + AA	221 (70.4)	337 (67.4)	Reference	
	Dominant model	CA + CC	258 (82.2)	386 (77.2)	1.361 (0.953–1.944)	0.090
		AA	56 (17.8)	114(22.8)	Reference	
	Genotype	TT	46 (14.6)	81 (16.2)	1.067 (0.694–1.642)	0.766
		TC	160 (51.0)	216 (43.2)	1.392 (1.021–1.899)	0.037
		CC	108 (34.4)	203 (40.6)	Reference	
rs9369217	Allele	T	252 (40.1)	378 (37.8)	1.103 (0.899–1.353)	0.348
		C	376 (59.9)	622 (62.2)	Reference	
	Recessive model	TT	46 (14.6)	81 (16.2)	0.888 (0.599–1.315)	0.553
		TC + CC	268 (85.4)	419 (83.8)	Reference	
	Dominant model	TC + TT	206 (65.6)	297 (59.4)	1.304 (0.972–1.748)	0.076
		CC	108 (34.4)	203 (40.6)	Reference	
	Genotype	TT	4(1.3)	7(1.6)	0.939 (0.272–3.243)	0.920
		TC	89 (28.3)	130 (26)	1.125 (0.819–1.544)	0.469
		CC	221 (70.4)	363 (72.6)	Reference	
	Allele	T	97(15.4)	144(14.4)	1.806 (0.821–1.436)	0.563
		C	531 (84.6)	856 (85.6)	Reference	
	Recessive model	TT	4(1.3)	7(1.6)	0.909 (0.264–3.130)	0.879
		TC + CC	310(98.7)	493 (98.4)	Reference	
	Dominant model	TC + TT	93 (29.6)	137(27.2)	1.115 (0.816–1.523)	0.494
		CC	221 (70.4)	363 (72.6)	Reference	

SLE, systemic lupus erythematosus; OR, odd ratio; 95% CI, 95% confidence interval.

### Relationship of *ET-1* gene polymorphisms with clinical, laboratory features in SLE patients

Association of qualitative and quantitative clinical, laboratory features in SLE patients with *ET-1* gene polymorphisms was shown in Supplementary table 7, 8, 9 and 10, respectively. Compared to patients without these clinical features, frequency of rs5070 genotypes TT + TG was elevated in patients with pericarditis and positive ANA (*P* = 0.034, *P* = 0.045), and was decreased in patients with fever (*P* = 0.005). Moreover, there was a higher frequency of allele T of rs5370 in patients with pericarditis, and a lower frequency of allele T of rs5370 in patients with pyuria compared with those in patients without the features (P = 0.013, *P* = 0.047). For rs1476046, decreased G allele frequency in patients with pericarditis and increased G allele frequency in patients with pyuria was observed when compared to patients without these features. A lower frequency of genotype GA of rs1476046 in SLE patients with fever and pyuria was noted (*P* = 0.013, *P* = 0.028). Interestingly, there were significant differences for genotypes and allele frequencies of rs2070699 polymorphism between SLE patients with alopecia and SLE patients without alopecia (*P* = 0.013, *P* = 0.035). Distribution of GG, GA, AA genotypes of rs2071942 and rs2071943 was different between SLE patients with and without fever (*P* = 0.025, *P* = 0.010). Patients with pericarditis had a declined frequency of G allele of rs2071942 and rs2071943 compared to patients without pericarditis (*P* = 0.019, *P* = 0.025). Frequency of G allele of rs2071942 and rs2071943 in patients with pyuria was higher (*P* = 0.035, *P* = 0.028). With respect to rs3087459, decreased frequencies of C allele and CC genotype in patients with positive ANA and increased frequency of CA genotype in patients with positive anti-SSB were observed when compared to patients without these features (*P* = 0.045, *P* = 0.029, *P* = 0.027). In addition, distribution of TT, TC and CC genotypes of rs9369217 was different in SLE patients with and without positive anti-SSB (*P* = 0.029) (Supplementary table 7 and 8). Other SNPs were not related to clinical and laboratory manifestations in SLE patients (Supplementary table 9).

When discussing quantitative indicators of clinical, laboratory features, levels of IgG between SLE patients with GG + GA genotype and AA genotype for rs5369 was different (*P* = 0.025). SLE patients carrying rs5369 AA genotype had higher levels of CRP as compared to the patients carrying GG + GA genotype (*P* = 0.012) (Supplementary table 10).

### *ET-1* gene haplotypes and SLE risk

**Figure 4:**
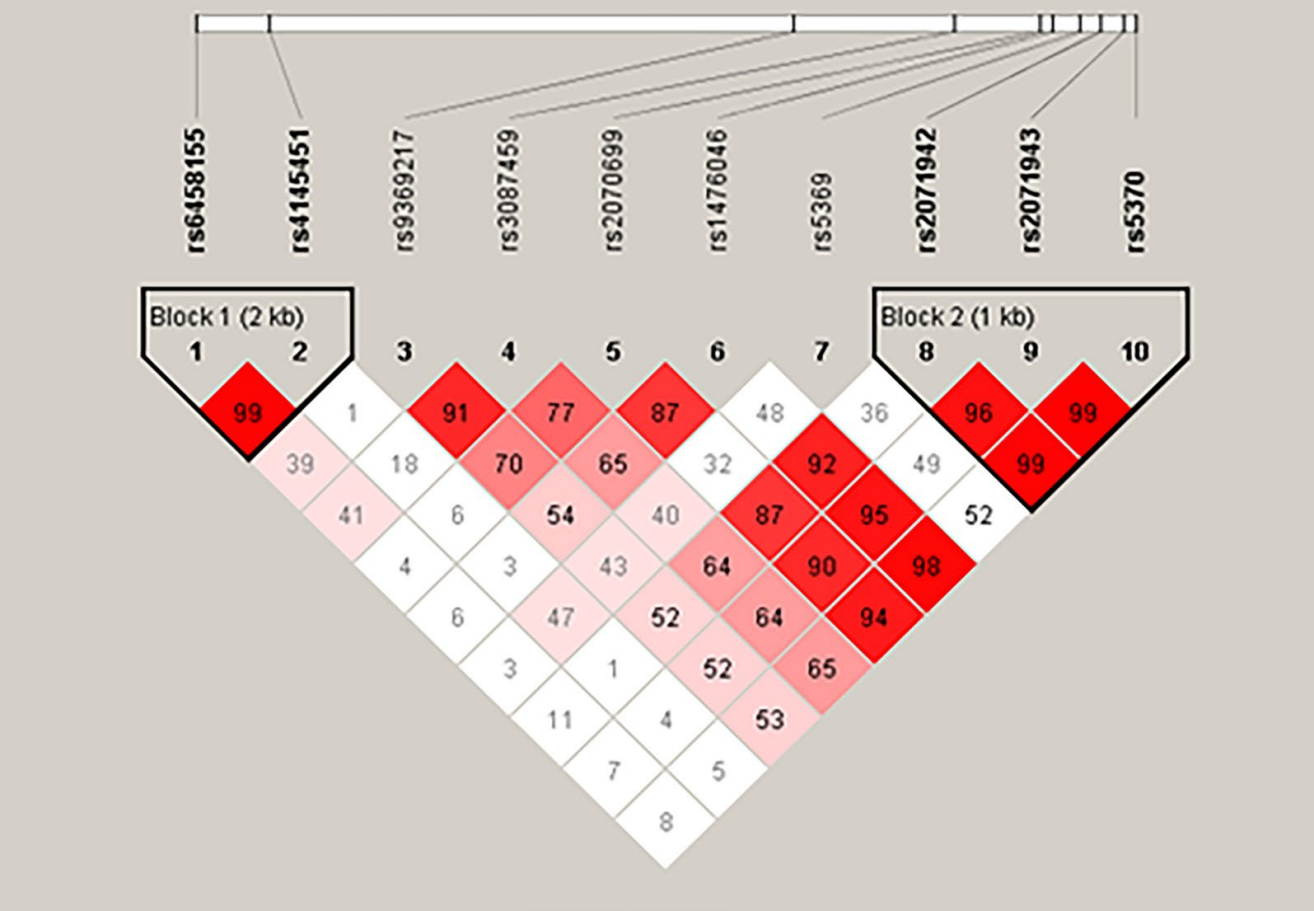
Linkage disequilibrium (LD) analysis for ten SNPs in* ET-1* gene. The color and numerical value (D’) of each box represent the Te intensity of LD. Red and pink indicate significant linkage, light blue and white indicate no linkage. The value of D’ varies from 0 to 1, by which value of 1 represents the maximum link. Block 1 consists of rs6458155 and rs4145451. Block 2 consists of rs2071942, rs2071943 and rs5370.

Considering genetic linkage disequilibrium, we analyzed correlation between *ET-1* gene haplotypes and SLE risk. There were two blocks constructed, one consisting of rs6458155, rs4145451 and the other one including rs2071942, rs2071943, rs5370 (Fig. 4). Results revealed that frequency of haplotype CA was lower in SLE patients compared to healthy controls (*P* = 0.012). Reduced frequency of haplotype AGG was found in patients with SLE (*P* = 0.041). No significant differences were observed in other haplotypes (Table 4).

**Table 4:** Haplotype analysis of *ET-1* gene polymorphisms between SLE and healthy controls.

Block*	Haplotype	Frequency	SLE ratio	Control ratio	χ^2^	*P* values
1	CC	0.551	0.556	0.548	0.093	0.761
	TA	0.385	0.398	0.377	0.723	0.395
	CA	0.062	0.043	0.074	6.351	0.012
2	GGG	0.691	0.674	0.701	1.399	0.237
	AAT	0.286	0.304	0.275	1.652	0.199
	AGG	0.016	0.008	0.021	4.179	0.041

*Block 1 consists of rs6458155 and rs4145451. Block 2 consists of rs2071942, rs2071943 and rs5370. SLE, systemic lupus erythematosus. SLE patient versus controls by 2 × 2 chi-square test.

## Discussion

SLE is a rheumatic disease with heterogeneous clinical symptoms that may result from excessive endothelial cells activation^16^. When activated by inflammatory stimuli, endothelial cells exhibit increased levels of surface adhesion molecules, which promote the migration and accumulation of leukocytes to the endothelial cells. Then, vascular obstruction and tissue hypoxia were induced, leading to apoptosis and tissue fibrosis^17^. Therefore, measuring biomarkers related to endothelial cells activation is useful for diagnosis of SLE. Endothelium-derived ET-1 plays a pathogenic role in connective tissue diseases, pulmonary hypertension, and cancer by affecting angiogenesis, inflammation, and fibrosis^18^. In OA mice, ET-1 induces increased levels of IL-18 through ET-1/ETAR axis and PI3K-dependent manner, promoting osteoblasts proliferation and exacerbating the disease^19^. As compared to control group, there were increased ET-1 levels in RA patients, especially in the patients with kidney and cardiovascular damages^20^. ET-1 levels were also elevated in other autoimmune diseases such as SSc, psoriasis and type 1 diabetes^21,22^. According to our study, serum ET-1 levels were significantly higher in SLE patients in training cohort when compared with healthy controls, which is consistent with previous findings^6,23,24^. Moreover, we found that serum ET-1 levels were associated with SLE patients complicated with cylindruria, alopecia and positive anti-Sm antibody. Similarly, Yoshio et al. showed that serum ET-1 in SLE patients correlated with IgM antibody levels. A study from Poland SLE patients reported that serum ET-1 levels were higher in active SLE patients than in inactive SLE patients and healthy controls^23^. SLE patients with visceral manifestation showed higher ET-1 levels as compared to that in patients without the feature^23^. In our study (the training cohort), we also found a positive correlation between serum ET-1 levels and SLEDAI score, and SLE patients with active disease activity had higher ET-1 levels. Urinary ET-1 may be a useful measurement of renal inflammatory activity and may serve as a marker in lupus nephritis disease activity^25^. In the present study, we also explored potential of serum ET-1 as a biomarker for SLE. In training cohort, AUC of ET-1 was 0.912 (95% CI 0.866–0.959), indicating that serum ET-1 could distinguish SLE patients from healthy subjects. In the validation cohort, ET-1 levels were higher in SLE patients than in other rheumatic diseases, including RA, OA, SS, AS, SSc. Compared with non-SLE diseases patients, AUC of serum ET-1 in SLE was higher than 0.800. Therefore, serum ET-1 is a promising marker for SLE. How ET-1 contributes to SLE or what mechanisms of ET-1 may involve in development of the disease? ET-1 could induce levels of connective tissue growth factor (CTGF/CCN2) by activating the MEK/ERK kinase pathway after binding to ETA and ETB receptor^26^. Indeed, these signalling pathways are pro-inflammatory pathways in SLE pathogenesis. After treatment with ET-1 receptor antagonist (bosentan), anti-dsDNA levels were reduced, glomerulosclerosis and renal T cells infiltration were improved in lupus mice^27^. These findings suggest that ET-1 may promote SLE progression. IL-17 and Th17 cells are important cytokine and cells in SLE pathogenesis. It is known that ET-1/ETAR signaling pathway regulates production of IL-17 in Th17 cells by an autocrine or paracrine manner^28^. To date, no study has discussed effects of ET-1 on Th17 cells in SLE. Thus, in future studies, we will consider to explore whether highly expressed ET-1 in SLE will contribute to lupus development by regulating Th17 cells. In patients with lupus nephritis, IgM anti-endothelial cell antibody and immune complexes can bind to endothelial cells in glomerular capillary rings and stimulate production of ET-1, leading to vascular injury^24^.

CCN3 involves in some autoimmune diseases pathogenesis^12^. Serum CCN3 levels were elevated in RA patients and positively correlated with levels of IL-6^29^. CCN3 levels were increased in SSc, multiple sclerosis patients^30,31^. For OA, CCN3 could inhibit PI3K/AKT/mTOR pathway by reducing HMGB1 levels and decrease extracellular matrix catabolism^32^. In our study, we found that serum CCN3 levels were significantly higher in SLE patients compared to normal subjects and were associated with thrombocytopenia. In addition, we observed a positive correlation between ET-1 levels and CCN3 levels in SLE patients. CCN3 inhibits levels of vascular adhesion molecules and reduces monocytes adhesion. CCN3 negatively regulates activation of NF-κB pathway, affecting inflammatory cytokines production of endothelial cells and cardiovascular homeostasis^33^. Type III interferons (IFNs) and type I IFNs may promote THP-1 cells differentiation, thereby contributing to the activation of follicular B cells and participating in development of autoimmune diseases^34^. IL-28B regulates innate and adaptive immune responses. SSc patients with pulmonary fibrosis had higher serum levels of IL-28B and *IL-28B* gene polymorphism (rs12979860) is associated with risk of pulmonary fibrosis in Caucasian SSc patients^35^. With respect to SLE, *IL-28B* gene SNPs (rs8099917, rs12979860) are risk factors for lupus nephritis in Taiwanese. Moreover, serum levels of IL-28B were elevated in SLE patients compared to healthy controls, which were related to complement levels and SLE disease activity^36^. IL-28B levels are associated with SLE disease activity^15^. According to our study, IL-28B levels in SLE patients were positively associated with disease activity and ESR levels. SLE patients with hematuria, proteinuria, cylindruria showed higher levels of IL-28B, which are typical clinical manifestations of SLE. We analyzed correlation between ET-1 and IL-28B levels and observed that serum levels of ET-1 were positively correlated with IL-28B levels. Therefore, in the future, we will conduct functional study (for example, knock out ET-1 gene in animal models) to discuss whether ET-1 regulates CCN3 or IL-28B, and then contributes to SLE development.

It is now accepted that SNPs are new genetic markers that may be used for discovery of high risk patients. *ET-1* gene polymorphisms are widely discussed in vascular-related diseases and cancer, such as hypertension, coronary atherosclerosis, and papillary thyroid cancer^37,38^. However, *ET-1* gene polymorphisms are less studied in autoimmune diseases. In this study, we explored relationship between *ET-1* gene polymorphisms and SLE risk by a case–control study from Chinese Han population. We found that genotypes of rs5370 (TG,TT + TG), rs1476046 (GG), rs2070699 (TT,TG), rs2071942 (GA), rs2071943 (GG), rs3087459 (CA + AA), rs6458155 (TC) and allele of rs2070699 (T) were associated with SLE susceptibility. For rs5370, TG, TT + TG genotype frequencies were higher in SLE patients, suggesting that rs5370 polymorphism may increase the risk of SLE. Frequency of rs2071942 GG genotype was increased in SLE patients compared to healthy subjects, which was positively associated with SLE risk. Mantaka et al. discussed association of *ET-1* gene rs2071942 and rs5370 polymorphisms with primary biliary cirrhosis (PBC), showing that distribution of genotypes and alleles of both loci were not significantly different between controls and PBC patients, whereas rs2071942 allele A and rs5370 allele T were associated with stage of disease progression^39^. These inconsistency may be due to differences in sample size, ethnicity, disease type. Similarly, frequencies of genotypes and alleles of rs5370 and rs18000541 were not significantly different between RA patients and healthy controls, but TT genotype of rs5370, rs18000541 was related to RA patients complicated with hypertension, indicating that rs5370 and rs18000541 loci were associated with cardiovascular risk in RA^40^. In our study, rs5370, rs1476046, rs2070699, rs2071942, rs2071943 rs5369, rs3087459, and rs9369217 polymorphisms correlated with some clinical, laboratory features in SLE patients. For example, SLE patients carrying rs2070699 T allele and TT genotype were more likely to develop symptoms of alopecia. Regarding rs2071942 and rs2071943, allele G was associated with pericarditis and pyuria, and genotype GA was related to fever. Moreover, significant correlation between rs5370 T allele, rs1476046 G allele and pericarditis, pyuria in SLE cases was observed. Rs3087459 (CC) and rs9369217 (TC) were associated with SLE patients with positive anti-SSB antibody. Rs5369 AA genotype correlated with IgG and CRP levels, suggesting that mutations at the rs5369 locus may affect levels of these disease markers in SLE patients. Indeed, CRP is an acute phase protein produced by hepatocytes in response to inflammation. CPR has important roles in active lupus nephritis. In SLE patients or mice models, IgG immune complexes are deposited in the spleen, causing damage to immune barrier of the spleen, and then numbers of autoantibodies are produced^41^. A study discussed association of graves’ disease (GD) risk and *ET-1* gene polymorphisms (rs5370 and rs1800541), revealing that the polymorphisms were not associated with disease susceptibility, but were related to autoantibodies production in GD patients^42^. To the best of our knowledge, this study is the first to discuss relationship between *ET-1* gene polymorphisms and SLE, which may offer new insights and basis for further discussion of *ET-1* genetic mutation and SLE in the future.

This study has several limitations. First, a larger number of SLE patients across multiple centers are required to confirm serum ET-1 as a potential disease marker for SLE. Second, the underlying mechanism by which ET-1 regulates CCN3, IL-28B, and subsequently influences pathogenesis of SLE requires further clarification.

In conclusion, ET-1 is associated with pathogenesis of SLE, may be a potential disease biomarker, and *ET-1* gene polymorphisms were related to SLE susceptibility in the Chinese Han population.

## Material and methods

### Subjects

This study included 314 SLE patients, 252 non-SLE patients, and 500 healthy controls. All patients were from Department of Rheumatology and Immunology of Affiliated Hospital of Southwest Medical University, and healthy controls came from Examination Center of Center for Disease Control and Prevention in Jiangyang District, Luzhou. SLE was diagnosed by 2019 American College of Rheumatology (ACR) revised criteria^43^. Healthy controls were the participants who did not have any diseases. Based on SLE disease activity index 2000 (SLEDAI-2 K) score^44^, SLE patients were divided into less active disease (SLEDIA < 10) and active disease (SLEDIA ≥ 10). RA, OA, SS, AS, SSc were diagnosed by 2010 ACR criteria for RA^45^, 1986 ACR criteria for OA^46^, 2016 ACR criteria for SS^47^, modifed New York criteria for AS^48^, 2013 ACR/European League Against Rheumatism criteria for SSc^49^, respectively. Participants were all Chinese Han population. This study was endorsed by Ethics Committee of Affiliated Hospital of Southwest Medical University and according to Declaration of Helsinki formulated by World Medical Association. Informed consent from each participant was obtained.

The study has two parts. In the first part, we evaluated serum levels of ET-1 in SLE patients, and discussed association of ET-1 with SLE pathogenesis, including 53 SLE patients and 80 healthy controls (training cohort). Similarly, we detected serum CCN3 and IL-28B levels in 53 SLE patients and 80 healthy individuals, and discussed correlation between ET-1 and CCN3, ET-1 and IL-28B. Then, another cohort of 102 SLE patients, 90 patients with RA, 95 with OA, 55 with SS, 38 with AS, and 17 with SSc, were included to discuss potential of serum ET-1 as a biomarker for SLE (validation cohort). In the second part, we genotyped 10 SNPs of *ET-1* gene (rs5369, rs5370, rs1476046, rs2070699, rs2071942, rs2071943, rs3087459, rs4145451, rs6458155, rs9369217) to discuss genetic susceptibility of SLE, including 314 SLE patients and 500 healthy controls.

### DNA preparation, screening and detection of single-nucleotide polymorphisms

Peripheral blood was centrifuged to obtain serum, which was stored at -80℃ until use. Genomic DNA was extracted by TIANamp Blood DNA kits (TIANGEN) on the basis of instructions. Ensemble database and 1000 genomes project (https://www.ncbi.nlm.nih.gov/variation/tools/1000genomes/) were used to find out information of *ET-1* gene polymorphisms in Chinese Han population. Criteria for SNP selection were as follows: (1) MAF > 0.05 in Chinese Han population. (2) SNPs were prior to be selected as they were located at functional positions such as 5’UTR, 3’UTR, frameshift mutation and non-synonymous mutation. *ET-1* gene polymorphisms (rs5369, rs5370, rs1476046, rs2070699, rs2071942, rs2071943, rs3087459, rs4145451, rs6458155, rs9369217) were genotyped using Kompetitive Allele-Specific PCR (KASP) method. Information of KASP primers for 10 SNPs was listed in Supplementary table 11. Briefly, KASP genotyping mix was prepared, consisting of 5ul DNA, 5ul 2 × KASP Master mix, and 0.14ul Assay mix. Then, the mix was added to PCR plates for PCR cycling reactions: (a) 94 °C (15 min); (b) 94 °C (20 s), 61 °C (60 s), for a total of 10 cycles; (c) 94 °C (20 s), 55 °C (60 s), for a total of 26 cycles. Finally, fluorescence data were read using an enzyme labeler with FRET function.

### Measurement of ET-1, CCN3, IL-28B by enzyme-linked immune sorbent assay (ELISA)

Levels of ET-1, CCN3 and IL-28B in SLE patients and control groups were determined by ELISA. All ELISA kits were purchased from CUSABIO (Wuhan, China, serial numbers CSB-E07007h (ET-1), CSB-EL015956HU (CCN3), CSB-E13296h (IL-28B)). According to the instructions, serum samples were added to the wells and incubated at 37 °C for 2 h. After removing the liquid, biotin-conjugate was added and incubated at 37 °C for 1 h. Subsequently, the plates were washed 3 times with washing buffer and HRP-avidin was added and incubated at 37 °C for 1 h. After 5 times’ washes, TMB substrate was added to each well and the termination solution was added after incubation at 37 °C for 15 min in the dark. The data were measured at 450 nm and converted into concentration by linear standard curve.

### Statistics

SPSS 23.0 and GraphPad Prism 5.01 were used for analysis of all data. For quantitative data, if the data followed normal distribution, means ± standard deviation (SD) and independent samples t-test were used to describe and analyze. Otherwise, median (interquartile range) and Wilcoxon ranks sum test were used. For categorical data, we described them by frequency and percentage, and adopted chi-square test for comparison. Potential of serum ET-1 as the biomarker for lupus was evaluated by area of receiver operating characteristic (ROC) curve. Hardy–Weinberg equilibrium assessed deviation of individual polymorphism in SLE patients and healthy controls. Distribution of genotypes and alleles between SLE patients and healthy controls was compared using chi-square test, then odds ratio (OR) and 95% confidence interval (CI) were analyzed by logistic regression model. HaploView 4.1 software was used to calculate linkage disequilibrium and haplotype analysis. The level of significance was *P* < 0.05 (two-sided).

### Supplementary Information

Supplementary Table 1.

Supplementary Table 2.

Supplementary Table 3.

Supplementary Table 4.

Supplementary Table 5.

Supplementary Table 6.

Supplementary Table 7.

Supplementary Table 8.

Supplementary Table 9.

Supplementary Table 10.

Supplementary Table 11.

## Data Availability

Datasets are available from the corresponding author on reasonable request.
