# Supplementary material for: Association between endothelin-1 and systemic lupus erythematosus: insights from a case–control study
Source: Sci Rep. 2023 Sep 25;13:15970. doi: 10.1038/s41598-023-43350-0 (PMC10520074; doi:10.1038/s41598-023-43350-0)
Supplement: Supplementary file 1 — Supplementary Table 1. [file 41598_2023_43350_MOESM1_ESM.docx]

Supplementary table 1 Correlation between serum levels of ET-1 with clinical features in SLE patients (qualitative variables).

| Clinical features | ET-1 (pg/ml) | Z | P value |
| --- | --- | --- | --- |
| Vasculitis |  |  |  |
| Positive | 165.29 (45.70-824.27) | -1.010 | 0.334 |
| Negative | 73.76 (40.00-112.64) |  |  |
| Arthritis |  |  |  |
| Positive | 56.13 (40.24-112.19) | 0.539 | 0.591 |
| Negative | 81.45 (38.50-143.77) |  |  |
| Rash |  |  |  |
| Positive | 82.50 (39.40-161.05) | -0.650 | 0.516 |
| Negative | 68.64 (40.24-105.97) |  |  |
| Alopecia |  |  |  |
| Positive | 105.97 (46.52-201.16) | -2.152 | 0.031 |
| Negative | 57.36 (36.76-104.08) |  |  |
| Pleurisy |  |  |  |
| Positive | 71.17 (47.40-92.48) | -0.224 | 0.836 |
| Negative | 74.11 (39.03-132.22) |  |  |
| Pericarditis |  |  |  |
| Positive | 45.34 (38.57-83.74) | 1.314 | 0.198 |
| Negative | 78.89 (40.12-143.77) |  |  |
| Fever |  |  |  |
| Positive | 113.10 (79.58-196.12) | -1.891 | 0.059 |
| Negative | 63.62 (38.50-107.52) |  |  |
| Hypocomplementemia |  |  |  |
| Positive | 71.38 (43.27-186.95) | -0.927 | 0.354 |
| Negative | 78.19 (36.43-112.19) |  |  |
| anti-dsDNA |  |  |  |
| Positive | 83.74 (36.86-105.97) | -0.178 | 0.859 |
| Negative | 71.20 (41.41-127.38) |  |  |
| Thrombocytopenia |  |  |  |
| Positive | 92.67 (45.52-183.90) | -1.491 | 0.136 |
| Negative | 63.62 (38.50-107.52) |  |  |
| Leukopenia |  |  |  |
| Positive | 97.96 (75.40-193.46) | -1.432 | 0.160 |
| Negative | 68.64 (38.57-113.10) |  |  |
| Hematuria |  |  |  |
| Positive | 92.67 (53.44-149.36) | -2.028 | 0.043 |
| Negative | 50.73 (36.14-95.01) |  |  |
| Proteinuria |  |  |  |
| Positive | 83.74 (53.58-149.36) | -2.708 | 0.007 |
| Negative | 42.28 (34.07-104.50) |  |  |
| Pyuria |  |  |  |
| Positive | 103.99 (32.80-244.77) |  | 0.859 |
| Negative | 74.11 (40.24-112.19) |  |  |
| Cylindruria |  |  |  |
| Positive | 115.57 (62.40-175.26) | -0.976 | 0.351 |
| Negative | 74.11 (39.16-112.64) |  |  |
| ANA |  |  |  |
| Positive | 78.19 (38.57-113.10) | -0.040 | 0.968 |
| Negative | 71.20 (41.41-137.88) |  |  |
| anti-Sm |  |  |  |
| Positive | 109.53 (72.84-199.92) | -2.945 | 0.003 |
| Negative | 46.52 (37.58-89.81) |  |  |
| anti-SSA |  |  |  |
| Positive | 81.66 (37.84-161.05) | -0.467 | 0.641 |
| Negative | 73.76 (41.80-104.29) |  |  |
| anti-SSB |  |  |  |
| Positive | 92.67 (39.76-149.36) | -0.548 | 0.583 |
| Negative | 73.93 (39.82-107.52) |  |  |
| anti-RNP |  |  |  |
| Positive | 78.19 (44.98-169.01) | -1.122 | 0.262 |
| Negative | 73.93 (38.37-110.21) |  |  |

SLE, systemic lupus erythematosustis; ANA, antinuclear antibody.
