# Supplementary material for: Association between endothelin-1 and systemic lupus erythematosus: insights from a case–control study
Source: Sci Rep. 2023 Sep 25;13:15970. doi: 10.1038/s41598-023-43350-0 (PMC10520074; doi:10.1038/s41598-023-43350-0)
Supplement: Supplementary file 2 — Supplementary Table 2. [file 41598_2023_43350_MOESM2_ESM.docx]

Supplementary table 2 Correlation between serum levels of ET-1 and SLE clinical features (quantitative variables).

| Clinical features | r_s_ | P value |
| --- | --- | --- |
| C3 | -0.245 | 0.086 |
| C4 | -0.175 | 0.234 |
| ESR | 0.479 | 0.005 |
| RF | -0.257 | 0.178 |
| IgA | 0.130 | 0.373 |
| IgM | 0.098 | 0.502 |
| IgG | 0.141 | 0.328 |
| CRP | -0.047 | 0.770 |
| SLEDAI | 0.463 | <0.001 |

SLE, systemic lupus erythematosustis; ESR, erythrocyte sedimentation rate; RF, rheumatoid factors; CRP, C-reactive protein; SLEDAI, systemic lupus erythematosus disease activity index.
