# Supplementary material for: Association between endothelin-1 and systemic lupus erythematosus: insights from a case–control study
Source: Sci Rep. 2023 Sep 25;13:15970. doi: 10.1038/s41598-023-43350-0 (PMC10520074; doi:10.1038/s41598-023-43350-0)
Supplement: Supplementary file 3 — Supplementary Table 3. [file 41598_2023_43350_MOESM3_ESM.docx]

Supplementary table 3 Correlation between serum levels of CCN3 with clinical features in SLE patients (qualitative variables).

| Clinical features | CCN3 (pg/ml) | Z | P value |
| --- | --- | --- | --- |
| Vasculitis |  |  |  |
| Positive | 875.49 (559.78-2623.53) | 0.876 | 0.405 |
| Negative | 733.67 (545.15-974.36) |  |  |
| Arthritis |  |  |  |
| Positive | 754.70 (393.95-1092.4) | -0.371 | 0.711 |
| Negative | 926.93 (590.32-912.64) |  |  |
| Rash |  |  |  |
| Positive | 673.91 (438.41-854.05) | -0.951 | 0.342 |
| Negative | 754.70 (587.30-988.08) |  |  |
| Alopecia |  |  |  |
| Positive | 738.83 (615.72-1092.42) | 1.364 | 0.172 |
| Negative | 677.93 (467.80-884.88) |  |  |
| Pleurisy |  |  |  |
| Positive | 787.77 (611.32-1057.52) | 1.006 | 0.315 |
| Negative | 688.52 (489.39-936.99) |  |  |
| Pericarditis |  |  |  |
| Positive | 679.83 (455.67-848.61) | -0.965 | 0.335 |
| Negative | 738.83 (586.11-990.44) |  |  |
| Fever |  |  |  |
| Positive | 733.67 (615.72-963.01) | 0.794 | 0.427 |
| Negative | 713.97 (467.80-971.37) |  |  |
| Hypocomplementemia |  |  |  |
| Positive | 738.83 (592.24-1027.58) | 1.793 | 0.073 |
| Negative | 592.15 (414.55-787.22) |  |  |
| ds-DNA |  |  |  |
| Positive | 858.86 (618.84-1126.93) | 1.718 | 0.086 |
| Negative | 608.77 (438.41-834.12) |  |  |
| Thrombocytopenia |  |  |  |
| Positive | 829.29 (738.83-1222.70) | 2.325 | 0.020 |
| Negative | 675.81 (453.00-936.99) |  |  |
| Leukopenia |  |  |  |
| Positive | 947.00 (659.12-1495.99) | 1.590 | 0.112 |
| Negative | 677.93 (489.39-942.66) |  |  |
| Hematuria |  |  |  |
| Positive | 770.10 (598.48-936.99) | 0.848 | 0.396 |
| Negative | 668.01 (446.44-1011.57) |  |  |
| Proteinuria |  |  |  |
| Positive | 736.25 (565.63-995.36) | 0.390 | 0.697 |
| Negative | 701.00 (418.74-990.44) |  |  |
| Pyuria |  |  |  |
| Positive | 768.58 (699.37-1333.93) | 0.082 | 0.400 |
| Negative | 690.42 (453.00-986.90) |  |  |
| Cylindruria |  |  |  |
| Positive | 739.40 (624.78-782.57) | -0.075 | 0.941 |
| Negative | 713.97 (467.80-989.26) |  |  |
| ANA |  |  |  |
| Positive | 679.83 (455.67-985.71) | -1.197 | 0.255 |
| Negative | 788.76 (577.25-1099.97) |  |  |
| anti-Sm |  |  |  |
| Positive | 733.67 (596.00-985.71) | 0.346 | 0.729 |
| Negative | 677.93 (460.58-983.58) |  |  |
| anti-SSA |  |  |  |
| Positive | 679.83 (590.32-974.36) | 0.515 | 0.607 |
| Negative | 667.41 (405.01-1092.19) |  |  |
| anti-SSB |  |  |  |
| Positive | 733.67 (601.82-928.31) | 0.687 | 0.492 |
| Negative | 673.91 (447.65-989.26) |  |  |
| anti-RNP |  |  |  |
| Positive | 642.97 (480.85-980.04) | -0.664 | 0.507 |
| Negative | 701.00 (586.11-1111.03) |  |  |

SLE, systemic lupus erythematosustis; ANA, antinuclear antibody.
