# Supplementary material for: Association between endothelin-1 and systemic lupus erythematosus: insights from a case–control study
Source: Sci Rep. 2023 Sep 25;13:15970. doi: 10.1038/s41598-023-43350-0 (PMC10520074; doi:10.1038/s41598-023-43350-0)
Supplement: Supplementary file 4 — Supplementary Table 4. [file 41598_2023_43350_MOESM4_ESM.docx]

Supplementary table 4 Correlation between serum levels of CCN3 and SLE clinical features (quantitative variables).

| Clinical features | r_s_ | P value |
| --- | --- | --- |
| C3 | -0.062 | 0.670 |
| C4 | -0.154 | 0.295 |
| ESR | 0.257 | 0.149 |
| RF | -0.013 | 0.945 |
| IgA | 0.051 | 0.730 |
| IgM | -0.041 | 0.779 |
| IgG | -0.094 | 0.517 |
| CRP | 0.118 | 0.464 |
| SLEDAI | 0.262 | 0.058 |

SLE, systemic lupus erythematosustis; ESR, erythrocyte sedimentation rate; RF, rheumatoid factors; CRP, C-reactive protein; SLEDAI, systemic lupus erythematosus disease activity index.
