# Supplementary material for: Association between endothelin-1 and systemic lupus erythematosus: insights from a case–control study
Source: Sci Rep. 2023 Sep 25;13:15970. doi: 10.1038/s41598-023-43350-0 (PMC10520074; doi:10.1038/s41598-023-43350-0)
Supplement: Supplementary file 5 — Supplementary Table 5. [file 41598_2023_43350_MOESM5_ESM.docx]

Supplementary table 5 Correlation between serum levels of IL-28B with clinical features in SLE patients (qualitative variable).

| Clinical features | IL-28B (pg/ml) | Z | P value |
| --- | --- | --- | --- |
| Vasculitis |  |  |  |
| Positive | 121.90 (30.28-920.15) | 1.577 | 0.115 |
| Negative | 22.88 (17.01-37.48) |  |  |
| Arthritis |  |  |  |
| Positive | 18.28 (15.48-24.97) | -2.015 | 0.052 |
| Negative | 28.02 (17.14-59.27) |  |  |
| Rash |  |  |  |
| Positive | 22.57 (15.01-65.88) | -0.454 | 0.650 |
| Negative | 23.70 (18.00-38.16) |  |  |
| Alopecia |  |  |  |
| Positive | 18.24 (16.85-31.49) | -1.283 | 0.200 |
| Negative | 25.18 (17.24-51.6) |  |  |
| Pleurisy |  |  |  |
| Positive | 31.40 (23.48-38.02) | 1.114 | 0.265 |
| Negative | 21.12 (16.55-38.71) |  |  |
| Pericarditis |  |  |  |
| Positive | 24.11 (17.01-39.53) | 0.164 | 0.780 |
| Negative | 22.10 (16.94-46.54) |  |  |
| Fever |  |  |  |
| Positive | 37.48 (28.02-77.06) | 1.863 | 0.063 |
| Negative | 21.32 (16.70-38.34) |  |  |
| Hypocomplementemia |  |  |  |
| Positive | 23.66 (16.97-50.96) | 0.285 | 0.775 |
| Negative | 24.11 (16.06-46.38) |  |  |
| anti-dsDNA |  |  |  |
| Positive | 23.30 (18.24-36.98) | 0.590 | 0.555 |
| Negative | 22.67 (16.62-41.94) |  |  |
| Thrombocytopenia |  |  |  |
| Positive | 43.56 (18.15-91.27) | 1.613 | 0.107 |
| Negative | 21.22 (16.62-33.00) |  |  |
| Leukopenia |  |  |  |
| Positive | 20.52 (1.40-99.93) | -0.458 | 0.647 |
| Negative | 23.98(17.0-37.98) |  |  |
| Hematuria |  |  |  |
| Positive | 36.98 (18.24-62.16) | 2.647 | 0.014 |
| Negative | 19.67 (15.01-30.73) |  |  |
| Proteinuria |  |  |  |
| Positive | 30.54 (18.28-56.38) | 2.500 | 0.012 |
| Negative | 17.24 (14.80-28.25) |  |  |
| Pyuria |  |  |  |
| Positive | 35.74 (24.01-165.18) | 1.769 | 0.077 |
| Negative | 22.89 (17.01-38.71) |  |  |
| Cylindruria |  |  |  |
| Positive | 69.25 (34.62-125.57) | 2.121 | 0.032 |
| Negative | 21.32 (16.70-38.09) |  |  |
| ANA |  |  |  |
| Positive | 24.10 (17.20-49.15) | 0.827 | 0.408 |
| Negative | 19.68 (16.08-38.16) |  |  |
| anti-Sm |  |  |  |
| Positive | 28.02 (17.28-56.38) | 0.635 | 0.525 |
| Negative | 21.14 (16.14-36.11) |  |  |
| anti-SSA |  |  |  |
| Positive | 25.18 (17.24-52.76) | 1.287 | 0.198 |
| Negative | 19.78 (14.94-27.97) |  |  |
| anti-SSB |  |  |  |
| Positive | 28.02 (18.90-100.52) | 1.560 | 0.119 |
| Negative | 22.67 (15.59-36.11) |  |  |
| anti-RNP |  |  |  |
| Positive | 20.11 (15.59-46.23) | -0.779 | 0.436 |
| Negative | 24.97 (18.24-39.53) |  |  |

SLE, systemic lupus erythematosustis; ANA, antinuclear antibody.
