# Supplementary material for: Association between endothelin-1 and systemic lupus erythematosus: insights from a case–control study
Source: Sci Rep. 2023 Sep 25;13:15970. doi: 10.1038/s41598-023-43350-0 (PMC10520074; doi:10.1038/s41598-023-43350-0)
Supplement: Supplementary file 6 — Supplementary Table 6. [file 41598_2023_43350_MOESM6_ESM.docx]

Supplementary table 6 Correlation between serum levels of IL-28B and SLE clinical features (quantitative variables).

| Clinical features | r_s_ | P value |
| --- | --- | --- |
| C3 | -0.143 | 0.327 |
| C4 | -0.104 | 0.481 |
| ESR | 0.461 | 0.007 |
| RF | -0.233 | 0.225 |
| IgA | -0.101 | 0.489 |
| IgM | -0.112 | 0.443 |
| IgG | 0.062 | 0.668 |
| CRP | 0.084 | 0.600 |
| SLEDAI | 0.406 | 0.003 |

SLE, systemic lupus erythematosustis; ESR, erythrocyte sedimentation rate; RF, rheumatoid factors; CRP, C-reactive protein; SLEDAI, systemic lupus erythematosus disease activity index.
