# Supplementary material for: Association between endothelin-1 and systemic lupus erythematosus: insights from a case–control study
Source: Sci Rep. 2023 Sep 25;13:15970. doi: 10.1038/s41598-023-43350-0 (PMC10520074; doi:10.1038/s41598-023-43350-0)
Supplement: Supplementary file 7 — Supplementary Table 7. [file 41598_2023_43350_MOESM7_ESM.docx]

| Clinical features | rs5370 | | | | | | | |  | | rs1476046 | | | | | | | |  | | rs2070699 | | | | | | | |  | | rs2071942 | | | | | | | | | |
| --- | --- | --- | --- | --- | --- | --- | --- | --- | --- | --- | --- | --- | --- | --- | --- | --- | --- | --- | --- | --- | --- | --- | --- | --- | --- | --- | --- | --- | --- | --- | --- | --- | --- | --- | --- | --- | --- | --- | --- | --- |
|  | Genotype frequency (n) | | | P1 | Allele frequency (n) | | P2 |  | | Genotype frequency (n) | | | | P1 | Allele frequency (n) | | P2 |  | | Genotype frequency (n) | | | | P1 | Allele frequency (n) | | P2 |  | | Genotype frequency (n) | | | | P1 | Allele frequency (n) | | | P2 | |  |
|  | TT | TG | GG |  | T | G |  |  | | GG | | GA | AA |  | G | A |  |  | | TT | | TG | GG |  | T | G |  |  | | GG | | GA | AA |  | G | A |  | |  |  |
| Lupus headache |  |  |  |  |  |  |  |  | |  | |  |  |  |  |  |  |  | |  | |  |  |  |  |  |  |  | |  | |  |  |  |  |  |  | |  |  |
| Positive | 3 | 6 | 12 | 0.918 | 12 | 30 | 0.788 |  | | 11 | | 7 | 3 | 0.411 | 29 | 13 | 0.691 |  | | 8 | | 6 | 7 | 0.286 | 22 | 20 | 0.685 |  | | 12 | | 5 | 4 | 0.070 | 29 | 13 | 0.970 | |  |  |
| Negative | 21 | 137 | 135 |  | 179 | 407 |  |  | | 123 | | 141 | 29 |  | 387 | 199 |  |  | | 77 | | 134 | 82 |  | 288 | 298 |  |  | | 134 | | 135 | 24 |  | 403 | 183 |  | |  |  |
| Vasculitis |  |  |  |  |  |  |  |  | |  | |  |  |  |  |  |  |  | |  | |  |  |  |  |  |  |  | |  | |  |  |  |  |  |  | |  |  |
| Positive | 2 | 14 | 12 | 0.884 | 18 | 38 | 0.768 |  | | 11 | | 15 | 2 | 0.729 | 37 | 19 | 0.977 |  | | 10 | | 8 | 10 | 0.201 | 28 | 28 | 0.920 |  | | 11 | | 13 | 4 | 0.506 | 35 | 21 | 0.287 | |  |  |
| Negative | 22 | 129 | 135 |  | 173 | 399 |  |  | | 123 | | 133 | 30 |  | 379 | 193 |  |  | | 75 | | 132 | 79 |  | 282 | 290 |  |  | | 135 | | 127 | 24 |  | 397 | 175 |  | |  |  |
| Arthritis |  |  |  |  |  |  |  |  | |  | |  |  |  |  |  |  |  | |  | |  |  |  |  |  |  |  | |  | |  |  |  |  |  |  | |  |  |
| Positive | 12 | 66 | 80 | 0.371 | 90 | 226 | 0.289 |  | | 72 | | 69 | 17 | 0.464 | 213 | 103 | 0.535 |  | | 46 | | 64 | 48 | 0.343 | 156 | 160 | 0.998 |  | | 79 | | 65 | 14 | 0.430 | 223 | 93 | 0.333 | |  |  |
| Negative | 12 | 77 | 67 |  | 101 | 211 |  |  | | 62 | | 79 | 15 |  | 203 | 109 |  |  | | 39 | | 76 | 41 |  | 154 | 158 |  |  | | 67 | | 75 | 14 |  | 209 | 103 |  | |  |  |
| Myositis |  |  |  |  |  |  |  |  | |  | |  |  |  |  |  |  |  | |  | |  |  |  |  |  |  |  | |  | |  |  |  |  |  |  | |  |  |
| Positive | 3 | 13 | 15 | 0.860 | 19 | 43 | 0.967 |  | | 12 | | 15 | 4 | 0.824 | 39 | 23 | 0.558 |  | | 6 | | 15 | 10 | 0.590 | 27 | 35 | 0.335 |  | | 14 | | 13 | 4 | 0.712 | 41 | 21 | 0.634 | |  |  |
| Negative | 21 | 130 | 132 |  | 172 | 394 |  |  | | 122 | | 133 | 28 |  | 377 | 189 |  |  | | 79 | | 125 | 79 |  | 283 | 283 |  |  | | 132 | | 127 | 24 |  | 391 | 175 |  | |  |  |
| Rash |  |  |  |  |  |  |  |  | |  | |  |  |  |  |  |  |  | |  | |  |  |  |  |  |  |  | |  | |  |  |  |  |  |  | |  |  |
| Positive | 7 | 58 | 67 | 0.284 | 72 | 192 | 0.145 |  | | 61 | | 61 | 10 | 0.327 | 183 | 81 | 0.165 |  | | 41 | | 54 | 37 | 0.361 | 136 | 128 | 0.358 |  | | 67 | | 57 | 8 | 0.215 | 191 | 73 | 0.101 | |  |  |
| Negative | 17 | 85 | 80 |  | 119 | 245 |  |  | | 73 | | 87 | 22 |  | 233 | 131 |  |  | | 44 | | 86 | 52 |  | 174 | 190 |  |  | | 79 | | 83 | 20 |  | 241 | 123 |  | |  |  |
| Alopecia |  |  |  |  |  |  |  |  | |  | |  |  |  |  |  |  |  | |  | |  |  |  |  |  |  |  | |  | |  |  |  |  |  |  | |  |  |
| Positive | 9 | 39 | 50 | 0.365 | 57 | 139 | 0.625 |  | | 47 | | 40 | 11 | 0.319 | 134 | 62 | 0.448 |  | | 37 | | 35 | 26 | 0.013 | 109 | 87 | 0.035 |  | | 50 | | 39 | 9 | 0.504 | 139 | 57 | 0.438 | |  |  |
| Negative | 15 | 104 | 97 |  | 134 | 298 |  |  | | 87 | | 108 | 21 |  | 282 | 150 |  |  | | 48 | | 105 | 63 |  | 201 | 231 |  |  | | 96 | | 101 | 19 |  | 293 | 139 |  | |  |  |
| Oral ulcer |  |  |  |  |  |  |  |  | |  | |  |  |  |  |  |  |  | |  | |  |  |  |  |  |  |  | |  | |  |  |  |  |  |  | |  |  |
| Positive | 4 | 17 | 24 | 0.528 | 25 | 65 | 0.557 |  | | 23 | | 16 | 6 | 0.237 | 62 | 28 | 0.566 |  | | 18 | | 17 | 10 | 0.106 | 53 | 37 | 0.051 |  | | 24 | | 17 | 4 | 0.584 | 65 | 25 | 0.448 | |  |  |
| Negative | 20 | 126 | 123 |  | 166 | 372 |  |  | | 111 | | 132 | 26 |  | 354 | 184 |  |  | | 67 | | 123 | 79 |  | 257 | 281 |  |  | | 122 | | 123 | 24 |  | 367 | 171 |  | |  |  |
| Pleurisy |  |  |  |  |  |  |  |  | |  | |  |  |  |  |  |  |  | |  | |  |  |  |  |  |  |  | |  | |  |  |  |  |  |  | |  |  |
| Positive | 1 | 13 | 11 | 0.682 | 15 | 35 | 0.947 |  | | 9 | | 14 | 2 | 0.649 | 32 | 18 | 0.727 |  | | 5 | | 15 | 5 | 0.270 | 25 | 25 | 0.925 |  | | 11 | | 13 | 1 | 0.575 | 35 | 15 | 0.847 | |  |  |
| Negative | 23 | 130 | 136 |  | 176 | 402 |  |  | | 125 | | 134 | 30 |  | 384 | 194 |  |  | | 80 | | 125 | 84 |  | 285 | 293 |  |  | | 135 | | 127 | 27 |  | 397 | 181 |  | |  |  |
| Pericarditis |  |  |  |  |  |  |  |  | |  | |  |  |  |  |  |  |  | |  | |  |  |  |  |  |  |  | |  | |  |  |  |  |  |  | |  |  |
| Positive | 4 | 15 | 6 | 0.034 | 23 | 27 | 0.013 |  | | 5 | | 16 | 4 | 0.055 | 26 | 24 | 0.026 |  | | 3 | | 13 | 9 | 0.204 | 19 | 31 | 0.094 |  | | 6 | | 15 | 4 | 0.052 | 27 | 23 | 0.019 | |  |  |
| Negative | 20 | 128 | 141 |  | 168 | 410 |  |  | | 129 | | 132 | 28 |  | 390 | 188 |  |  | | 82 | | 127 | 80 |  | 291 | 287 |  |  | | 140 | | 125 | 24 |  | 405 | 173 |  | |  |  |
| Fever |  |  |  |  |  |  |  |  | |  | |  |  |  |  |  |  |  | |  | |  |  |  |  |  |  |  | |  | |  |  |  |  |  |  | |  |  |
| Positive | 10 | 20 | 31 | 0.005 | 40 | 82 | 0.526 |  | | 27 | | 22 | 12 | 0.013 | 76 | 46 | 0.304 |  | | 14 | | 27 | 20 | 0.605 | 55 | 67 | 0.292 |  | | 31 | | 20 | 10 | 0.025 | 82 | 40 | 0.675 | |  |  |
| Negative | 14 | 123 | 116 |  | 151 | 355 |  |  | | 107 | | 126 | 20 |  | 340 | 166 |  |  | | 71 | | 113 | 69 |  | 255 | 251 |  |  | | 115 | | 120 | 18 |  | 350 | 156 |  | |  |  |
| Hypocomplementemia | |  |  |  |  |  |  |  | |  | |  |  |  |  |  |  |  | |  | |  |  |  |  |  |  |  | |  | |  |  |  |  |  |  | |  |  |
| Positive | 11 | 78 | 68 | 0.338 | 100 | 214 | 0.435 |  | | 64 | | 82 | 11 | 0.077 | 210 | 104 | 0.736 |  | | 44 | | 70 | 43 | 0.902 | 158 | 156 | 0.632 |  | | 67 | | 64 | 14 | 0.913 | 198 | 92 | 0.797 | |  |  |
| Negative | 13 | 65 | 79 |  | 91 | 223 |  |  | | 70 | | 66 | 21 |  | 206 | 108 |  |  | | 41 | | 70 | 46 |  | 152 | 162 |  |  | | 79 | | 76 | 14 |  | 234 | 104 |  | |  |  |
| ds-DNA |  |  |  |  |  |  |  |  | |  | |  |  |  |  |  |  |  | |  | |  |  |  |  |  |  |  | |  | |  |  |  |  |  |  | |  |  |
| Positive | 5 | 30 | 35 | 0.832 | 40 | 100 | 0.591 |  | | 33 | | 31 | 6 | 0.668 | 97 | 43 | 0.388 |  | | 18 | | 30 | 22 | 0.809 | 66 | 74 | 0.551 |  | | 35 | | 29 | 6 | 0.798 | 99 | 41 | 0.577 | |  |  |
| Negative | 19 | 113 | 112 |  | 151 | 337 |  |  | | 101 | | 117 | 26 |  | 319 | 169 |  |  | | 67 | | 110 | 67 |  | 244 | 244 |  |  | | 111 | | 111 | 22 |  | 333 | 155 |  | |  |  |
| Thrombocytopenia |  |  |  |  |  |  |  |  | |  | |  |  |  |  |  |  |  | |  | |  |  |  |  |  |  |  | |  | |  |  |  |  |  |  | |  |  |
| Positive | 6 | 22 | 19 | 0.301 | 34 | 60 | 0.188 |  | | 17 | | 23 | 7 | 0.410 | 57 | 37 | 0.213 |  | | 10 | | 24 | 13 | 0.545 | 44 | 50 | 0.591 |  | | 18 | | 22 | 7 | 0.215 | 58 | 36 | 0.108 | |  |  |
| Negative | 18 | 121 | 128 |  | 157 | 377 |  |  | | 117 | | 125 | 25 |  | 359 | 175 |  |  | | 75 | | 116 | 76 |  | 266 | 268 |  |  | | 128 | | 118 | 21 |  | 374 | 160 |  | |  |  |
| Leukopenia |  |  |  |  |  |  |  |  | |  | |  |  |  |  |  |  |  | |  | |  |  |  |  |  |  |  | |  | |  |  |  |  |  |  | |  |  |
| Positive | 2 | 18 | 16 | 0.795 | 22 | 50 | 0.978 |  | | 16 | | 18 | 2 | 0.619 | 50 | 22 | 0.541 |  | | 13 | | 13 | 10 | 0.389 | 39 | 33 | 0.386 |  | | 16 | | 18 | 2 | 0.664 | 50 | 22 | 0.899 | |  |  |
| Negative | 22 | 125 | 131 |  | 169 | 387 |  |  | | 118 | | 130 | 30 |  | 366 | 190 |  |  | | 72 | | 127 | 79 |  | 271 | 285 |  |  | | 130 | | 122 | 26 |  | 382 | 174 |  | |  |  |
| Hematuria |  |  |  |  |  |  |  |  | |  | |  |  |  |  |  |  |  | |  | |  |  |  |  |  |  |  | |  | |  |  |  |  |  |  | |  |  |
| Positive | 8 | 49 | 50 | 0.996 | 65 | 149 | 0.987 |  | | 47 | | 52 | 8 | 0.520 | 146 | 68 | 0.450 |  | | 27 | | 48 | 32 | 0.842 | 102 | 112 | 0.540 |  | | 50 | | 49 | 8 | 0.805 | 149 | 65 | 0.745 | |  |  |
| Negative | 16 | 94 | 97 |  | 126 | 288 |  |  | | 87 | | 96 | 24 |  | 270 | 144 |  |  | | 58 | | 92 | 57 |  | 208 | 206 |  |  | | 96 | | 91 | 20 |  | 283 | 131 |  | |  |  |
| Proteinuria |  |  |  |  |  |  |  |  | |  | |  |  |  |  |  |  |  | |  | |  |  |  |  |  |  |  | |  | |  |  |  |  |  |  | |  |  |
| Positive | 12 | 61 | 81 | 0.105 | 85 | 223 | 0.132 |  | | 73 | | 67 | 14 | 0.248 | 213 | 95 | 0.130 |  | | 42 | | 73 | 39 | 0.469 | 157 | 151 | 0.428 |  | | 80 | | 59 | 15 | 0.840 | 219 | 89 | 0.219 | |  |  |
| Negative | 12 | 82 | 66 |  | 106 | 214 |  |  | | 61 | | 81 | 18 |  | 203 | 117 |  |  | | 43 | | 67 | 50 |  | 153 | 167 |  |  | | 66 | | 81 | 13 |  | 213 | 107 |  | |  |  |
| Pyuria |  |  |  |  |  |  |  |  | |  | |  |  |  |  |  |  |  | |  | |  |  |  |  |  |  |  | |  | |  |  |  |  |  |  | |  |  |
| Positive | 1 | 8 | 18 | 0.094 | 10 | 44 | 0.047 |  | | 18 | | 8 | 1 | 0.028 | 44 | 10 | 0.013 |  | | 9 | | 12 | 6 | 0.664 | 30 | 24 | 0.341 |  | | 18 | | 8 | 1 | 0.083 | 44 | 10 | 0.035 | |  |  |
| Negative | 23 | 135 | 129 |  | 181 | 393 |  |  | | 116 | | 140 | 31 |  | 372 | 202 |  |  | | 76 | | 128 | 83 |  | 280 | 294 |  |  | | 128 | | 132 | 27 |  | 388 | 186 |  | |  |  |
| Cylindruria |  |  |  |  |  |  |  |  | |  | |  |  |  |  |  |  |  | |  | |  |  |  |  |  |  |  | |  | |  |  |  |  |  |  | |  |  |
| Positive | 7 | 7 | 2 | 0.754 | 21 | 11 | 0.617 |  | | 7 | | 7 | 2 | 0.935 | 21 | 11 | 0.940 |  | | 6 | | 6 | 4 | 0.699 | 18 | 14 | 0.514 |  | | 7 | | 6 | 3 | 0.360 | 20 | 12 | 0.431 | |  |  |
| Negative | 140 | 136 | 22 |  | 416 | 180 |  |  | | 127 | | 141 | 30 |  | 395 | 201 |  |  | | 83 | | 134 | 81 |  | 300 | 296 |  |  | | 139 | | 134 | 25 |  | 412 | 184 |  | |  |  |
| ANA |  |  |  |  |  |  |  |  | |  | |  |  |  |  |  |  |  | |  | |  |  |  |  |  |  |  | |  | |  |  |  |  |  |  | |  |  |
| Positive | 82 | 79 | 7 | 0.045 | 243 | 93 | 0.110 |  | | 75 | | 82 | 11 | 0.072 | 232 | 104 | 0.111 |  | | 49 | | 70 | 49 | 0.506 | 168 | 168 | 0.732 |  | | 82 | | 77 | 9 | 0.059 | 241 | 95 | 0.088 | |  |  |
| Negative | 65 | 64 | 17 |  | 194 | 98 |  |  | | 59 | | 66 | 21 |  | 184 | 108 |  |  | | 40 | | 70 | 36 |  | 150 | 142 |  |  | | 64 | | 63 | 19 |  | 191 | 101 |  | |  |  |
| anti-Sm |  |  |  |  |  |  |  |  | |  | |  |  |  |  |  |  |  | |  | |  |  |  |  |  |  |  | |  | |  |  |  |  |  |  | |  |  |
| Positive | 32 | 40 | 4 | 0.313 | 104 | 48 | 0.720 |  | | 29 | | 44 | 3 | 0.034 | 102 | 50 | 0.796 |  | | 28 | | 30 | 18 | 0.168 | 86 | 66 | 0.092 |  | | 32 | | 40 | 4 | 0.185 | 104 | 48 | 0.910 | |  |  |
| Negative | 115 | 103 | 20 |  | 333 | 143 |  |  | | 105 | | 104 | 29 |  | 314 | 162 |  |  | | 61 | | 110 | 67 |  | 232 | 244 |  |  | | 114 | | 100 | 24 |  | 328 | 148 |  | |  |  |
| anti-SSA |  |  |  |  |  |  |  |  | |  | |  |  |  |  |  |  |  | |  | |  |  |  |  |  |  |  | |  | |  |  |  |  |  |  | |  |  |
| Positive | 61 | 61 | 8 | 0.692 | 183 | 77 | 0.715 |  | | 56 | | 62 | 12 | 0.894 | 174 | 86 | 0.762 |  | | 35 | | 56 | 39 | 0.614 | 126 | 134 | 0.359 |  | | 61 | | 59 | 10 | 0.813 | 181 | 79 | 0.707 | |  |  |
| Negative | 86 | 82 | 16 |  | 254 | 114 |  |  | | 78 | | 86 | 20 |  | 242 | 126 |  |  | | 54 | | 84 | 46 |  | 192 | 176 |  |  | | 85 | | 81 | 18 |  | 251 | 117 |  | |  |  |
| anti-SSB |  |  |  |  |  |  |  |  | |  | |  |  |  |  |  |  |  | |  | |  |  |  |  |  |  |  | |  | |  |  |  |  |  |  | |  |  |
| Positive | 17 | 26 | 2 | 0.188 | 60 | 30 | 0.515 |  | | 16 | | 27 | 2 | 0.124 | 59 | 31 | 0.882 |  | | 12 | | 22 | 11 | 0.817 | 46 | 44 | 0.923 |  | | 17 | | 24 | 4 | 0.415 | 58 | 32 | 0.336 | |  |  |
| Negative | 130 | 117 | 22 |  | 377 | 161 |  |  | | 118 | | 121 | 30 |  | 357 | 181 |  |  | | 77 | | 118 | 74 |  | 272 | 266 |  |  | | 129 | | 116 | 24 |  | 374 | 164 |  | |  |  |
| anti-RNP |  |  |  |  |  |  |  |  | |  | |  |  |  |  |  |  |  | |  | |  |  |  |  |  |  |  | |  | |  |  |  |  |  |  | |  |  |
| Positive | 43 | 46 | 6 | 0.729 | 132 | 58 | 0.968 |  | | 39 | | 49 | 7 | 0.423 | 127 | 63 | 0.834 |  | | 28 | | 42 | 25 | 0.954 | 98 | 92 | 0.756 |  | | 43 | | 44 | 8 | 0.917 | 130 | 60 | 0.895 | |  |  |
| Negative | 104 | 97 | 18 |  | 305 | 133 |  |  | | 95 | | 99 | 25 |  | 289 | 149 |  |  | | 61 | | 98 | 60 |  | 220 | 218 |  |  | | 103 | | 96 | 20 |  | 302 | 136 |  | |  |  |
| anti-Jo-1 |  |  |  |  |  |  |  |  | |  | |  |  |  |  |  |  |  | |  | |  |  |  |  |  |  |  | |  | |  |  |  |  |  |  | |  |  |
| Positive | 1 | 0 | 0 | 0.566 | 2 | 0 | 0.349 |  | | 1 | | 0 | 0 | 0.51 | 2 | 0 | 0.312 |  | | 0 | | 0 | 1 | 0.259 | 0 | 2 | 0.151 |  | | 1 | | 0 | 0 | 0.561 | 2 | 0 | 0.340 | |  |  |
| Negative | 146 | 143 | 24 |  | 435 | 191 |  |  | | 133 | | 148 | 32 |  | 414 | 212 |  |  | | 89 | | 140 | 84 |  | 318 | 308 |  |  | | 145 | | 140 | 28 |  | 430 | 196 |  | |  |  |

Supplementary table 7 Association of *ET-1* gene polymorphisms (rs5370, rs1476046, rs2070699 and rs2071942) with clinical features in SLE patients (qualitative variables, positive results).

SLE, systemic lupus erythematosustis; ANA, antinuclear antibody.
