# Supplementary material for: Association between endothelin-1 and systemic lupus erythematosus: insights from a case–control study
Source: Sci Rep. 2023 Sep 25;13:15970. doi: 10.1038/s41598-023-43350-0 (PMC10520074; doi:10.1038/s41598-023-43350-0)
Supplement: Supplementary file 8 — Supplementary Table 8. [file 41598_2023_43350_MOESM8_ESM.docx]

| Clinical features | rs2071943 | | | | | | |  | rs3087459 | | | | | | |  | rs9369217 | | | | | | | |
| --- | --- | --- | --- | --- | --- | --- | --- | --- | --- | --- | --- | --- | --- | --- | --- | --- | --- | --- | --- | --- | --- | --- | --- | --- |
|  | Genotype frequency (n) | | | P1 | Allele frequency (n) | | P2 |  | Genotype frequency (n) | | | P1 | Allele frequency (n) | | P2 |  | Genotype frequency (n) | | | P1 | Allele frequency (n) | | P2 |  |
|  | GG | GA | AA |  | G | A |  |  | CC | CA | AA |  | C | A |  |  | TT | TC | CC |  | T | C |  |  |
| Lupus headache |  |  |  |  |  |  |  |  |  |  |  |  |  |  |  |  |  |  |  |  |  |  |  |  |
| Positive | 12 | 6 | 3 | 0.264 | 30 | 12 | 0.637 |  | 1 | 5 | 15 | 0.247 | 7 | 35 | 0.329 |  | 0 | 3 | 18 | 0.272 | 3 | 39 | 0.123 |  |
| Negative | 131 | 136 | 26 |  | 398 | 188 |  |  | 7 | 122 | 164 |  | 136 | 450 |  |  | 4 | 86 | 203 |  | 94 | 492 |  |  |
| Vasculitis |  |  |  |  |  |  |  |  |  |  |  |  |  |  |  |  |  |  |  |  |  |  |  |  |
| Positive | 12 | 14 | 2 | 0.840 | 38 | 18 | 0.960 |  | 1 | 11 | 16 | 0.934 | 13 | 43 | 0.934 |  | 1 | 9 | 18 | 0.451 | 11 | 45 | 0.362 |  |
| Negative | 131 | 128 | 27 |  | 390 | 182 |  |  | 7 | 116 | 163 |  | 130 | 442 |  |  | 3 | 80 | 203 |  | 86 | 486 |  |  |
| Arthritis |  |  |  |  |  |  |  |  |  |  |  |  |  |  |  |  |  |  |  |  |  |  |  |  |
| Positive | 77 | 66 | 15 | 0.456 | 220 | 96 | 0.427 |  | 2 | 66 | 90 | 0.335 | 70 | 246 | 0.710 |  | 1 | 43 | 114 | 0.519 | 45 | 271 | 0.400 |  |
| Negative | 66 | 76 | 14 |  | 208 | 104 |  |  | 6 | 61 | 89 |  | 73 | 239 |  |  | 3 | 46 | 107 |  | 52 | 260 |  |  |
| Myositis |  |  |  |  |  |  |  |  |  |  |  |  |  |  |  |  |  |  |  |  |  |  |  |  |
| Positive | 15 | 13 | 3 | 0.927 | 43 | 19 | 0.831 |  | 0 | 9 | 22 | 0.206 | 9 | 53 | 0.103 |  | 0 | 6 | 25 | 0.380 | 6 | 56 | 0.186 |  |
| Negative | 128 | 129 | 26 |  | 385 | 181 |  |  | 8 | 118 | 157 |  | 134 | 432 |  |  | 4 | 83 | 196 |  | 91 | 475 |  |  |
| Rash |  |  |  |  |  |  |  |  |  |  |  |  |  |  |  |  |  |  |  |  |  |  |  |  |
| Positive | 65 | 58 | 9 | 0.331 | 188 | 76 | 0.161 |  | 4 | 50 | 78 | 0.686 | 58 | 206 | 0.684 |  | 3 | 37 | 92 | 0.405 | 43 | 221 | 0.619 |  |
| Negative | 78 | 84 | 20 |  | 240 | 124 |  |  | 4 | 77 | 101 |  | 85 | 279 |  |  | 1 | 52 | 129 |  | 54 | 310 |  |  |
| Alopecia |  |  |  |  |  |  |  |  |  |  |  |  |  |  |  |  |  |  |  |  |  |  |  |  |
| Positive | 49 | 39 | 10 | 0.429 | 137 | 59 | 0.527 |  | 4 | 39 | 55 | 0.509 | 47 | 149 | 0.627 |  | 2 | 27 | 69 | 0.708 | 31 | 165 | 0.863 |  |
| Negative | 94 | 103 | 19 |  | 291 | 141 |  |  | 4 | 88 | 124 |  | 96 | 336 |  |  | 2 | 62 | 152 |  | 66 | 366 |  |  |
| Oral ulcer |  |  |  |  |  |  |  |  |  |  |  |  |  |  |  |  |  |  |  |  |  |  |  |  |
| Positive | 24 | 17 | 4 | 0.509 | 65 | 25 | 0.371 |  | 0 | 14 | 31 | 0.152 | 14 | 76 | 0.078 |  | 0 | 9 | 36 | 0.266 | 9 | 81 | 0.122 |  |
| Negative | 119 | 125 | 25 |  | 363 | 175 |  |  | 8 | 113 | 148 |  | 129 | 409 |  |  | 4 | 80 | 185 |  | 88 | 450 |  |  |
| Pleurisy |  |  |  |  |  |  |  |  |  |  |  |  |  |  |  |  |  |  |  |  |  |  |  |  |
| Positive | 11 | 13 | 1 | 0.578 | 35 | 15 | 0.770 |  | 0 | 12 | 13 | 0.550 | 12 | 38 | 0.829 |  | 0 | 7 | 18 | 0.836 | 7 | 43 | 0.768 |  |
| Negative | 132 | 129 | 28 |  | 393 | 185 |  |  | 8 | 115 | 166 |  | 131 | 447 |  |  | 4 | 82 | 203 |  | 90 | 488 |  |  |
| Pericarditis |  |  |  |  |  |  |  |  |  |  |  |  |  |  |  |  |  |  |  |  |  |  |  |  |
| Positive | 6 | 15 | 4 | 0.066 | 27 | 23 | 0.025 |  | 1 | 14 | 10 | 0.199 | 16 | 34 | 0.105 |  | 0 | 8 | 17 | 0.780 | 8 | 42 | 0.910 |  |
| Negative | 137 | 127 | 25 |  | 401 | 177 |  |  | 7 | 113 | 169 |  | 127 | 451 |  |  | 4 | 81 | 204 |  | 89 | 489 |  |  |
| Fever |  |  |  |  |  |  |  |  |  |  |  |  |  |  |  |  |  |  |  |  |  |  |  |  |
| Positive | 30 | 20 | 11 | 0.010 | 80 | 42 | 0.496 |  | 1 | 26 | 34 | 0.837 | 28 | 94 | 0.958 |  | 0 | 12 | 49 | 0.133 | 12 | 110 | 0.056 |  |
| Negative | 113 | 122 | 18 |  | 348 | 158 |  |  | 7 | 101 | 145 |  | 115 | 391 |  |  | 4 | 77 | 172 |  | 85 | 421 |  |  |
| Hypocomplementemia | |  |  |  |  |  |  |  |  |  |  |  |  |  |  |  |  |  |  |  |  |  |  |  |
| Positive | 67 | 65 | 13 | 0.972 | 199 | 91 | 0.816 |  | 3 | 74 | 80 | 0.050 | 80 | 234 | 0.106 |  | 2 | 47 | 108 | 0.821 | 51 | 263 | 0.581 |  |
| Negative | 76 | 77 | 16 |  | 229 | 109 |  |  | 5 | 53 | 99 |  | 63 | 251 |  |  | 2 | 42 | 113 |  | 46 | 268 |  |  |
| ds-DNA |  |  |  |  |  |  |  |  |  |  |  |  |  |  |  |  |  |  |  |  |  |  |  |  |
| Positive | 34 | 30 | 6 | 0.845 | 98 | 42 | 0.595 |  | 1 | 34 | 35 | 0.261 | 36 | 104 | 0.346 |  | 2 | 22 | 46 | 0.308 | 26 | 114 | 0.246 |  |
| Negative | 109 | 112 | 23 |  | 330 | 158 |  |  | 7 | 93 | 144 |  | 107 | 381 |  |  | 2 | 67 | 175 |  | 71 | 417 |  |  |
| Thrombocytopenia |  |  |  |  |  |  |  |  |  |  |  |  |  |  |  |  |  |  |  |  |  |  |  |  |
| Positive | 19 | 21 | 7 | 0.327 | 59 | 35 | 0.224 |  | 1 | 22 | 24 | 0.627 | 24 | 70 | 0.489 |  | 0 | 18 | 29 | 0.199 | 18 | 76 | 0.281 |  |
| Negative | 124 | 121 | 22 |  | 369 | 165 |  |  | 7 | 105 | 155 |  | 119 | 415 |  |  | 4 | 71 | 192 |  | 79 | 455 |  |  |
| Leukopenia |  |  |  |  |  |  |  |  |  |  |  |  |  |  |  |  |  |  |  |  |  |  |  |  |
| Positive | 16 | 18 | 2 | 0.666 | 50 | 22 | 0.803 |  | 0 | 17 | 19 | 0.444 | 17 | 55 | 0.857 |  | 0 | 12 | 24 | 0.620 | 12 | 60 | 0.761 |  |
| Negative | 127 | 124 | 27 |  | 378 | 178 |  |  | 8 | 110 | 160 |  | 126 | 430 |  |  | 4 | 77 | 197 |  | 85 | 471 |  |  |
| Hematuria |  |  |  |  |  |  |  |  |  |  |  |  |  |  |  |  |  |  |  |  |  |  |  |  |
| Positive | 49 | 49 | 9 | 0.935 | 147 | 67 | 0.835 |  | 4 | 39 | 64 | 0.413 | 47 | 167 | 0.728 |  | 1 | 27 | 79 | 0.614 | 29 | 185 | 0.345 |  |
| Negative | 94 | 93 | 20 |  | 281 | 133 |  |  | 4 | 88 | 115 |  | 96 | 318 |  |  | 3 | 62 | 142 |  | 68 | 346 |  |  |
| Proteinuria |  |  |  |  |  |  |  |  |  |  |  |  |  |  |  |  |  |  |  |  |  |  |  |  |
| Positive | 80 | 60 | 14 | 0.069 | 220 | 88 | 0.084 |  | 3 | 61 | 90 | 0.745 | 67 | 241 | 0.551 |  | 1 | 43 | 110 | 0.609 | 45 | 263 | 0.570 |  |
| Negative | 63 | 82 | 15 |  | 208 | 112 |  |  | 5 | 66 | 89 |  | 76 | 244 |  |  | 3 | 46 | 111 |  | 52 | 268 |  |  |
| Pyuria |  |  |  |  |  |  |  |  |  |  |  |  |  |  |  |  |  |  |  |  |  |  |  |  |
| Positive | 18 | 8 | 1 | 0.065 | 44 | 10 | 0.028 |  | 1 | 6 | 20 | 0.129 | 8 | 46 | 0.145 |  | 0 | 7 | 20 | 0.781 | 7 | 47 | 0.597 |  |
| Negative | 125 | 134 | 28 |  | 384 | 190 |  |  | 7 | 121 | 159 |  | 135 | 439 |  |  | 4 | 82 | 201 |  | 90 | 484 |  |  |
| Cylindruria |  |  |  |  |  |  |  |  |  |  |  |  |  |  |  |  |  |  |  |  |  |  |  |  |
| Positive | 7 | 7 | 2 | 0.898 | 21 | 11 | 0.753 |  | 0 | 6 | 10 | 0.758 | 6 | 26 | 0.578 |  | 13 | 3 | 0 | 0.593 | 29 | 3 | 0.329 |  |
| Negative | 136 | 135 | 27 |  | 407 | 189 |  |  | 8 | 121 | 169 |  | 137 | 459 |  |  | 208 | 86 | 4 |  | 502 | 94 |  |  |
| ANA |  |  |  |  |  |  |  |  |  |  |  |  |  |  |  |  |  |  |  |  |  |  |  |  |
| Positive | 80 | 79 | 9 | 0.039 | 239 | 97 | 0.086 |  | 1 | 64 | 103 | 0.029 | 66 | 270 | 0.045 |  | 122 | 44 | 2 | 0.648 | 288 | 48 | 0.388 |  |
| Negative | 63 | 63 | 20 |  | 189 | 103 |  |  | 7 | 63 | 76 |  | 77 | 215 |  |  | 99 | 45 | 2 |  | 243 | 49 |  |  |
| anti-Sm |  |  |  |  |  |  |  |  |  |  |  |  |  |  |  |  |  |  |  |  |  |  |  |  |
| Positive | 32 | 40 | 4 | 0.203 | 104 | 48 | 0.935 |  | 2 | 34 | 40 | 0.672 | 38 | 114 | 0.452 |  | 52 | 22 | 2 | 0.468 | 126 | 26 | 0.516 |  |
| Negative | 111 | 102 | 25 |  | 324 | 152 |  |  | 6 | 93 | 139 |  | 105 | 371 |  |  | 169 | 67 | 2 |  | 405 | 71 |  |  |
| anti-SSA |  |  |  |  |  |  |  |  |  |  |  |  |  |  |  |  |  |  |  |  |  |  |  |  |
| Positive | 60 | 61 | 9 | 0.486 | 181 | 79 | 0.508 |  | 2 | 55 | 73 | 0.575 | 59 | 201 | 0.969 |  | 90 | 38 | 2 | 0.893 | 218 | 42 | 0.680 |  |
| Negative | 83 | 81 | 20 |  | 247 | 121 |  |  | 6 | 72 | 106 |  | 84 | 284 |  |  | 131 | 51 | 2 |  | 313 | 55 |  |  |
| anti-SSB |  |  |  |  |  |  |  |  |  |  |  |  |  |  |  |  |  |  |  |  |  |  |  |  |
| Positive | 17 | 26 | 2 | 0.147 | 60 | 30 | 0.744 |  | 0 | 26 | 19 | 0.027 | 26 | 64 | 0.135 |  | 25 | 20 | 0 | 0.029 | 70 | 20 | 0.055 |  |
| Negative | 126 | 116 | 27 |  | 368 | 170 |  |  | 8 | 101 | 160 |  | 117 | 421 |  |  | 196 | 69 | 4 |  | 461 | 77 |  |  |
| anti-RNP |  |  |  |  |  |  |  |  |  |  |  |  |  |  |  |  |  |  |  |  |  |  |  |  |
| Positive | 43 | 46 | 6 | 0.457 | 132 | 58 | 0.640 |  | 1 | 44 | 50 | 0.245 | 46 | 144 | 0.571 |  | 63 | 32 | 0 | 0.180 | 158 | 32 | 0.524 |  |
| Negative | 100 | 96 | 23 |  | 296 | 142 |  |  | 7 | 83 | 129 |  | 97 | 341 |  |  | 158 | 57 | 4 |  | 373 | 65 |  |  |
| anti-Jo-1 |  |  |  |  |  |  |  |  |  |  |  |  |  |  |  |  |  |  |  |  |  |  |  |  |
| Positive | 1 | 0 | 0 | 0.549 | 2 | 0 | 0.333 |  | 0 | 0 | 1 | 0.685 | 0 | 2 | 0.442 |  | 1 | 0 | 0 | 0.810 | 2 | 0 | 0.545 |  |
| Negative | 142 | 142 | 29 |  | 426 | 200 |  |  | 8 | 127 | 178 |  | 143 | 483 |  |  | 220 | 89 | 4 |  | 529 | 97 |  |  |

Supplementary table 8 Association of *ET-1* gene polymorphisms (rs2071943, rs3087459 and rs9369217) with clinical features in SLE patients (qualitative variables, positive results).

SLE, systemic lupus erythematosustis; ANA, antinuclear antibody.
