# Supplementary material for: Association between endothelin-1 and systemic lupus erythematosus: insights from a case–control study
Source: Sci Rep. 2023 Sep 25;13:15970. doi: 10.1038/s41598-023-43350-0 (PMC10520074; doi:10.1038/s41598-023-43350-0)
Supplement: Supplementary file 9 — Supplementary Table 9. [file 41598_2023_43350_MOESM9_ESM.docx]

| Clinical features | rs5369 | | | | | | |  | rs4145451 | | | | | | | |  | | rs6458155 | | | | | | | |
| --- | --- | --- | --- | --- | --- | --- | --- | --- | --- | --- | --- | --- | --- | --- | --- | --- | --- | --- | --- | --- | --- | --- | --- | --- | --- | --- |
|  | Genotype frequency (n) | | | P1 | Allele frequency (n) | | P2 |  | Genotype frequency (n) | | | P1 | Allele frequency (n) | | P2 |  | | Genotype frequency (n) | | | | P1 | Allele frequency (n) | | P2 |  |
|  | GG | GA | AA |  | G | A |  |  | CC | CA | AA |  | C | A |  |  |  | TT | | TC | CC |  | T | C |  |  |
| Lupus headache |  |  |  |  |  |  |  |  |  |  |  |  |  |  |  |  | |  | |  |  |  |  |  |  |  |
| Positive | 19 | 1 | 1 | 0.484 | 39 | 3 | 0.347 |  | 5 | 11 | 5 | 0.702 | 21 | 21 | 0.426 |  | | 4 | | 10 | 7 | 0.838 | 18 | 24 | 0.709 |  |
| Negative | 273 | 16 | 4 |  | 562 | 24 |  |  | 88 | 154 | 51 |  | 330 | 256 |  |  | | 42 | | 150 | 101 |  | 234 | 352 |  |  |
| Vasculitis |  |  |  |  |  |  |  |  |  |  |  |  |  |  |  |  | |  | |  |  |  |  |  |  |  |
| Positive | 28 | 0 | 0 | 0.314 | 56 | 0 | 0.097 |  | 5 | 16 | 7 | 0.295 | 26 | 30 | 0.135 |  | | 6 | | 16 | 6 | 0.260 | 28 | 28 | 0.114 |  |
| Negative | 264 | 17 | 5 |  | 545 | 27 |  |  | 88 | 149 | 49 |  | 325 | 247 |  |  | | 40 | | 144 | 102 |  | 224 | 348 |  |  |
| Arthritis |  |  |  |  |  |  |  |  |  |  |  |  |  |  |  |  | |  | |  |  |  |  |  |  |  |
| Positive | 148 | 7 | 3 | 0.680 | 303 | 13 | 0.818 |  | 43 | 83 | 32 | 0.435 | 169 | 147 | 0.221 |  | | 27 | | 82 | 49 | 0.301 | 136 | 180 | 0.134 |  |
| Negative | 144 | 10 | 2 |  | 298 | 14 |  |  | 50 | 82 | 24 |  | 182 | 130 |  |  | | 19 | | 78 | 59 |  | 116 | 196 |  |  |
| Myositis |  |  |  |  |  |  |  |  |  |  |  |  |  |  |  |  | |  | |  |  |  |  |  |  |  |
| Positive | 31 | 0 | 0 | 0.274 | 62 | 0 | 0.079 |  | 5 | 19 | 7 | 0.218 | 29 | 33 | 0.128 |  | | 5 | | 18 | 8 | 0.568 | 28 | 34 | 0.394 |  |
| Negative | 261 | 17 | 5 |  | 539 | 27 |  |  | 88 | 146 | 49 |  | 322 | 244 |  |  | | 41 | | 142 | 100 |  | 224 | 342 |  |  |
| Rash |  |  |  |  |  |  |  |  |  |  |  |  |  |  |  |  | |  | |  |  |  |  |  |  |  |
| Positive | 121 | 8 | 3 | 0.644 | 250 | 14 | 0.291 |  | 47 | 63 | 22 | 0.140 | 157 | 107 | 0.124 |  | | 18 | | 63 | 51 | 0.403 | 99 | 165 | 0.253 |  |
| Negative | 171 | 9 | 2 |  | 351 | 13 |  |  | 46 | 102 | 34 |  | 194 | 170 |  |  | | 28 | | 97 | 57 |  | 153 | 211 |  |  |
| Alopecia |  |  |  |  |  |  |  |  |  |  |  |  |  |  |  |  | |  | |  |  |  |  |  |  |  |
| Positive | 92 | 4 | 2 | 0.719 | 188 | 8 | 0.856 |  | 29 | 48 | 21 | 0.502 | 106 | 90 | 0.538 |  | | 18 | | 46 | 34 | 0.407 | 82 | 114 | 0.556 |  |
| Negative | 200 | 13 | 3 |  | 413 | 19 |  |  | 64 | 117 | 35 |  | 245 | 187 |  |  | | 28 | | 114 | 74 |  | 170 | 262 |  |  |
| Oral ulcer |  |  |  |  |  |  |  |  |  |  |  |  |  |  |  |  | |  | |  |  |  |  |  |  |  |
| Positive | 44 | 1 | 0 | 0.376 | 89 | 1 | 0.107 |  | 9 | 25 | 11 | 0.221 | 43 | 47 | 0.094 |  | | 10 | | 22 | 13 | 0.278 | 42 | 48 | 0.171 |  |
| Negative | 248 | 16 | 5 |  | 512 | 26 |  |  | 84 | 140 | 45 |  | 308 | 230 |  |  | | 36 | | 138 | 95 |  | 210 | 328 |  |  |
| Pleurisy |  |  |  |  |  |  |  |  |  |  |  |  |  |  |  |  | |  | |  |  |  |  |  |  |  |
| Positive | 24 | 1 | 0 | 0.756 | 49 | 1 | 0.403 |  | 7 | 13 | 5 | 0.953 | 27 | 23 | 0.779 |  | | 4 | | 14 | 7 | 0.782 | 22 | 28 | 0.560 |  |
| Negative | 268 | 16 | 5 |  | 552 | 26 |  |  | 86 | 152 | 51 |  | 324 | 254 |  |  | | 42 | | 146 | 101 |  | 230 | 348 |  |  |
| Pericarditis |  |  |  |  |  |  |  |  |  |  |  |  |  |  |  |  | |  | |  |  |  |  |  |  |  |
| Positive | 25 | 0 | 0 | 0.359 | 50 | 0 | 0.118 |  | 3 | 17 | 5 | 0.125 | 23 | 27 | 0.142 |  | | 4 | | 18 | 3 | 0.042 | 26 | 24 | 0.074 |  |
| Negative | 267 | 17 | 5 |  | 551 | 27 |  |  | 90 | 148 | 51 |  | 328 | 250 |  |  | | 42 | | 142 | 105 |  | 226 | 352 |  |  |
| Fever |  |  |  |  |  |  |  |  |  |  |  |  |  |  |  |  | |  | |  |  |  |  |  |  |  |
| Positive | 57 | 3 | 1 | 0.982 | 117 | 5 | 0.903 |  | 17 | 36 | 8 | 0.443 | 70 | 52 | 0.713 |  | | 8 | | 29 | 24 | 0.659 | 45 | 77 | 0.416 |  |
| Negative | 235 | 14 | 4 |  | 484 | 22 |  |  | 76 | 129 | 48 |  | 281 | 225 |  |  | | 38 | | 131 | 84 |  | 207 | 299 |  |  |
| Hypocomplementemia | |  |  |  |  |  |  |  |  |  |  |  |  |  |  |  | |  | |  |  |  |  |  |  |  |
| Positive | 146 | 7 | 4 | 0.312 | 299 | 15 | 0.555 |  | 45 | 89 | 23 | 0.234 | 179 | 135 | 0.574 |  | | 20 | | 79 | 56 | 0.636 | 119 | 191 | 0.380 |  |
| Negative | 146 | 10 | 1 |  | 302 | 12 |  |  | 48 | 76 | 33 |  | 172 | 142 |  |  | | 26 | | 81 | 52 |  | 133 | 185 |  |  |
| ds-DNA |  |  |  |  |  |  |  |  |  |  |  |  |  |  |  |  | |  | |  |  |  |  |  |  |  |
| Positive | 66 | 3 | 1 | 0.975 | 135 | 5 | 0.630 |  | 22 | 36 | 12 | 0.930 | 80 | 60 | 0.735 |  | | 11 | | 34 | 25 | 0.898 | 56 | 84 | 0.972 |  |
| Negative | 226 | 14 | 4 |  | 466 | 22 |  |  | 71 | 129 | 44 |  | 271 | 217 |  |  | | 35 | | 126 | 83 |  | 196 | 292 |  |  |
| Thrombocytopenia |  |  |  |  |  |  |  |  |  |  |  |  |  |  |  |  | |  | |  |  |  |  |  |  |  |
| Positive | 45 | 2 | 0 | 0.588 | 92 | 2 | 0.260 |  | 11 | 31 | 5 | 0.121 | 53 | 41 | 0.917 |  | | 5 | | 27 | 15 | 0.559 | 37 | 57 | 0.870 |  |
| Negative | 247 | 15 | 5 |  | 509 | 25 |  |  | 82 | 134 | 51 |  | 298 | 236 |  |  | | 41 | | 133 | 93 |  | 215 | 319 |  |  |
| Leukopenia |  |  |  |  |  |  |  |  |  |  |  |  |  |  |  |  | |  | |  |  |  |  |  |  |  |
| Positive | 34 | 1 | 1 | 0.641 | 69 | 3 | 0.953 |  | 10 | 20 | 6 | 0.929 | 40 | 32 | 0.951 |  | | 4 | | 18 | 14 | 0.743 | 26 | 46 | 0.460 |  |
| Negative | 258 | 16 | 4 |  | 532 | 24 |  |  | 83 | 145 | 50 |  | 311 | 245 |  |  | | 42 | | 142 | 94 |  | 226 | 330 |  |  |
| Hematuria |  |  |  |  |  |  |  |  |  |  |  |  |  |  |  |  | |  | |  |  |  |  |  |  |  |
| Positive | 101 | 6 | 0 | 0.268 | 208 | 6 | 0.184 |  | 35 | 53 | 19 | 0.669 | 123 | 91 | 0.565 |  | | 16 | | 52 | 39 | 0.824 | 84 | 130 | 0.748 |  |
| Negative | 191 | 11 | 5 |  | 393 | 21 |  |  | 58 | 112 | 37 |  | 228 | 186 |  |  | | 30 | | 108 | 69 |  | 168 | 246 |  |  |
| Proteinuria |  |  |  |  |  |  |  |  |  |  |  |  |  |  |  |  | |  | |  |  |  |  |  |  |  |
| Positive | 144 | 8 | 2 | 0.905 | 296 | 12 | 0.625 |  | 46 | 76 | 32 | 0.356 | 168 | 140 | 0.505 |  | | 27 | | 73 | 54 | 0.286 | 127 | 181 | 0.579 |  |
| Negative | 148 | 9 | 3 |  | 305 | 15 |  |  | 47 | 89 | 24 |  | 183 | 137 |  |  | | 19 | | 87 | 54 |  | 125 | 195 |  |  |
| Pyuria |  |  |  |  |  |  |  |  |  |  |  |  |  |  |  |  | |  | |  |  |  |  |  |  |  |
| Positive | 26 | 1 | 0 | 0.717 | 53 | 1 | 0.354 |  | 7 | 13 | 7 | 0.514 | 27 | 27 | 0.362 |  | | 4 | | 15 | 8 | 0.853 | 23 | 31 | 0.699 |  |
| Negative | 266 | 16 | 5 |  | 548 | 26 |  |  | 86 | 152 | 49 |  | 324 | 250 |  |  | | 42 | | 145 | 100 |  | 229 | 345 |  |  |
| Cylindruria |  |  |  |  |  |  |  |  |  |  |  |  |  |  |  |  | |  | |  |  |  |  |  |  |  |
| Positive | 16 | 0 | 0 | 0.530 | 32 | 0 | 0.281 |  | 5 | 9 | 2 | 0.849 | 19 | 13 | 0.684 |  | | 8 | | 6 | 2 | 0.397 | 22 | 10 | 0.293 |  |
| Negative | 276 | 17 | 5 |  | 569 | 27 |  |  | 88 | 156 | 54 |  | 332 | 264 |  |  | | 100 | | 154 | 44 |  | 354 | 242 |  |  |
| ANA |  |  |  |  |  |  |  |  |  |  |  |  |  |  |  |  | |  | |  |  |  |  |  |  |  |
| Positive | 159 | 7 | 2 | 0.470 | 325 | 11 | 0.174 |  | 48 | 90 | 30 | 0.902 | 186 | 150 | 0.772 |  | | 53 | | 92 | 23 | 0.349 | 198 | 138 | 0.605 |  |
| Negative | 133 | 10 | 3 |  | 276 | 16 |  |  | 45 | 75 | 26 |  | 165 | 127 |  |  | | 55 | | 68 | 23 |  | 178 | 114 |  |  |
| anti-Sm |  |  |  |  |  |  |  |  |  |  |  |  |  |  |  |  | |  | |  |  |  |  |  |  |  |
| Positive | 72 | 3 | 1 | 0.787 | 147 | 5 | 0.481 |  | 22 | 43 | 11 | 0.619 | 87 | 65 | 0.701 |  | | 28 | | 40 | 8 | 0.498 | 96 | 56 | 0.343 |  |
| Negative | 220 | 14 | 4 |  | 454 | 22 |  |  | 71 | 122 | 45 |  | 264 | 212 |  |  | | 80 | | 120 | 38 |  | 280 | 196 |  |  |
| anti-SSA |  |  |  |  |  |  |  |  |  |  |  |  |  |  |  |  | |  | |  |  |  |  |  |  |  |
| Positive | 120 | 8 | 2 | 0.887 | 248 | 12 | 0.743 |  | 35 | 72 | 23 | 0.642 | 142 | 118 | 0.588 |  | | 42 | | 73 | 15 | 0.232 | 157 | 103 | 0.826 |  |
| Negative | 172 | 9 | 3 |  | 353 | 15 |  |  | 58 | 93 | 33 |  | 209 | 159 |  |  | | 66 | | 87 | 31 |  | 219 | 149 |  |  |
| anti-SSB |  |  |  |  |  |  |  |  |  |  |  |  |  |  |  |  | |  | |  |  |  |  |  |  |  |
| Positive | 42 | 3 | 0 | 0.610 | 87 | 3 | 0.625 |  | 10 | 24 | 11 | 0.323 | 44 | 46 | 0.148 |  | | 12 | | 25 | 8 | 0.477 | 49 | 41 | 0.256 |  |
| Negative | 250 | 14 | 5 |  | 514 | 24 |  |  | 83 | 141 | 45 |  | 307 | 231 |  |  | | 96 | | 135 | 38 |  | 327 | 211 |  |  |
| anti-RNP |  |  |  |  |  |  |  |  |  |  |  |  |  |  |  |  | |  | |  |  |  |  |  |  |  |
| Positive | 90 | 4 | 1 | 0.720 | 184 | 6 | 0.353 |  | 33 | 51 | 11 | 0.121 | 117 | 73 | 0.059 |  | | 37 | | 50 | 8 | 0.105 | 124 | 66 | 0.069 |  |
| Negative | 202 | 13 | 4 |  | 417 | 21 |  |  | 60 | 114 | 45 |  | 234 | 204 |  |  | | 71 | | 110 | 38 |  | 252 | 186 |  |  |
| anti-Jo-1 |  |  |  |  |  |  |  |  |  |  |  |  |  |  |  |  | |  | |  |  |  |  |  |  |  |
| Positive | 1 | 0 | 0 | 0.963 | 2 | 0 | 0.764 |  | 0 | 1 | 0 | 0.636 | 1 | 1 | 0.867 |  | | 0 | | 1 | 0 | 0.617 | 1 | 1 | 0.775 |  |
| Negative | 291 | 17 | 5 |  | 599 | 27 |  |  | 93 | 164 | 56 |  | 350 | 276 |  |  | | 108 | | 159 | 46 |  | 375 | 251 |  |  |

Supplementary table 9 Association of *ET-1* gene polymorphisms (rs5369, rs4145451 and rs6458155) with clinical features in SLE patients (qualitative variables, negative results).

SLE, systemic lupus erythematosustis; ANA, antinuclear antibody.
