# Supplementary material for: Association between endothelin-1 and systemic lupus erythematosus: insights from a case–control study
Source: Sci Rep. 2023 Sep 25;13:15970. doi: 10.1038/s41598-023-43350-0 (PMC10520074; doi:10.1038/s41598-023-43350-0)
Supplement: Supplementary file 10 — Supplementary Table 10. [file 41598_2023_43350_MOESM10_ESM.docx]

Supplementary table 10 Association of *ET-1* gene polymorphisms with clinical features in SLE patients (quantitative variables).

| Characteristics | rs5369 [Median (P_25_-P_75_)] | | | P^*^ | rs5370 [Median (P_25_-P_75_)] | | | P^*^ |
| --- | --- | --- | --- | --- | --- | --- | --- | --- |
|  | GG | GA | AA |  | TT | TG | GG |  |
| C3 (g/L） | 0.72 (0.45-0.93) | 0.96 (0.61-1.17) | 0.64 (0.47-0.70) | 0.422 | 0.72 (0.44-0.89) | 0.69 (0.44-0.97) | 0.79 (0.50-0.95) | 0.296 |
| C4 (g/L) | 0.15 (0.07-0.22) | 0.13 (0.07-0.25) | 0.10 (0.05-0.16) | 0.316 | 0.17 (0.06-0.22) | 0.13 (0.07-0.21) | 0.15 (0.08-0.25) | 0.238 |
| ESR (mm/H) | 26.00 (11.00-51.00) | 16.15 (7.00-36.50) | –^**^ | 0.831 | 44 (13.75-63.75) | 23.00 (8.25-51.00) | 26.5 (11-48.25) | 0.948 |
| RF (IU/ml) | 9.70 (8.45-12.85) | 8.25 (6.65-10.03) | –^**^ | 0.717 | 9.40 (3.78-42.63) | 10.90 (8.40-18.00) | 9.70 (7.25-11.00) | 0.073 |
| IgA (mg/L) | 2.45 (1.84-3.30) | 2.24 (1.56-3.95) | 3.06 (1.60-8.51) | 0.634 | 3.09 (1.98-3.42) | 2.57 (1.77-3.35) | 2.48 (1.84-3.28) | 0.564 |
| IgM (mg/L) | 1.01 (0.70-1.50) | 0.64 (0.34-0.86) | 1.17 (0.71-1.38) | 0.865 | 1.23 (0.86-1.93) | 1.05 (0.75-1.40) | 0.91 (0.52-1.52) | 0.327 |
| IgG (g/L) | 14.24 (10.57-19.45) | 12.86 (9.44-20.24) | 21.85 (17.71-49.35) | 0.025 | 18.17 (13.28-21.08) | 14.37 (10.97-20.81) | 13.76 (10.73-18.86) | 0.236 |
| CRP (mg/L) | 3.01 (0.55-15.17) | 3.40 (0.50-13.83) | 0.43 (0.16-0.50) | 0.012 | 3.45 (1.20-35.70) | 2.951 (0.50-15.65) | 2.12 (0.50-12.61) | 0.378 |
| Characteristics | rs1476046 [Median (P_25_-P_75_)] | | | P^*^ | rs2070699 [Median (P_25_-P_75_)] | | | P^*^ |
|  | TT | TG | GG |  | TT | TG | GG |  |
| C3 (g/L） | 0.76 (0.50-0.94) | 0.69 (0.43-0.95) | 0.82 (0.52-0.95) | 0.351 | 0.73 (0.48-0.94) | 0.75 (0.44-0.94) | 0.70 (0.50-0.97) | 0.674 |
| C4 (g/L) | 0.15 (0.07-0.24) | 0.13 (0.07-0.21) | 0.18 (0.08-0.23) | 0.493 | 0.15 (0.08-0.24) | 0.13 (0.07-0.21) | 0.16 (0.66-0.23) | 0.59 |
| ESR (mm/H) | 28.00 (11-47.50) | 23.00 (10-55) | 34.00 (12.5-62.25) | 0.349 | 28.00 (13.00-50.00) | 23.50 (9.75-41.50) | 33.00 (12.00-60.00) | 0.367 |
| RF (IU/ml) | 9.70 (7.65-10.90) | 10.15 (8.85-14.05) | 31.65 (7.33-722.23) | 0.298 | 10.00 (7.33-11.08) | 9.70 (8.10-11.00) | 10.10 (8.75-18.40) | 0.156 |
| IgA (mg/L) | 2.49 (1.88-3.56) | 2.60 (1.73-3.34) | 2.66 (1.89-3.48) | 0.556 | 2.54 (2.01-3.26) | 2.50 (1.68-3.12) | 2.61 (1.77-3.51) | 0.299 |
| IgM (mg/L) | 1.05 (0.62-1.51) | 1.00 (0.75-1.27) | 1.12 (0.81-1.91) | 0.232 | 0.98 (0.68-1.55) | 0.97 (0.61-1.41) | 1.06 (0.77-1.43) | 0.304 |
| IgG (g/L) | 13.89 (10.54-19.10) | 14.25 (10.55-20.62) | 15.88 (11.03-19.90) | 0.502 | 15.83 (11.43-20.30) | 12.68 (9.87-19.08) | 15.30 (10.89-20.49) | 0.228 |
| CRP (mg/L) | 2.75 (0.51-13.18) | 3.00 (0.5-15.08) | 3.30 (0.93-21.48) | 0.445 | 1.80 (0.50-12.78) | 3.00 (0.50-13.27) | 3.30 (0.54-25.65) | 0.465 |
| Characteristics | rs2071942 [Median (P_25_-P_75_)] | | | P^*^ | rs2071943 [Median (P_25_-P_75_)] | | | P^*^ |
|  | GG | GA | AA |  | GG | GA | AA |  |
| C3 (g/L） | 0.79 (0.50-0.95) | 0.68 (0.44-0.97) | 0.74 (0.43-0.86) | 0.569 | 0.79 (0.50-0.94) | 0.69 (0.44-0.97) | 0.76 (0.48-0.90) | 0.918 |
| C4 (g/L) | 0.15 (0.08-0.24) | 0.13 (0.07-0.21) | 0.18 (0.06-0.23) | 0.919 | 0.15 (0.07-0.24) | 0.13 (0.07-0.21) | 0.18 (0.08-0.23) | 0.770 |
| ESR (mm/H) | 27.00 (12.00-47.50) | 23.00 (10.00-55.00) | 34.00 (11.25-62.25) | 0.292 | 26.50 (11.50-48.25) | 23.00 (10.50-54.00) | 35.00 (10.00-63.00) | 0.296 |
| RF (IU/ml) | 9.70 (8.00-11.00) | 10.10 (8.70-14.10) | 9.70 (5.55-36.20) | 0.851 | 9.70 (8.00-11.00) | 10.10 (8.70-14.10) | 9.40 (5.75-49.63) | 0.781 |
| IgA (mg/L) | 2.49 (1.82-3.28) | 2.58 (1.77-3.35) | 2.97 (2.12-3.45) | 0.211 | 2.51 (1.84-3.28) | 2.58 (1.78-3.33) | 2.97 (1.89-3.45) | 0.334 |
| IgM (mg/L) | 0.99 (0.62-1.52) | 1.03 (0.75-1.32) | 1.06 (0.80-1.82) | 0.315 | 0.97 (0.63-1.52) | 1.03 (0.75-1.32) | 1.12 (0.80-1.72) | 0.312 |
| IgG (g/L) | 13.63 (10.40-18.99) | 14.37 (10.97-20.81) | 16.83 (10.84-20.45) | 0.279 | 13.52 (10.05-18.86) | 14.48 (11.11-20.80) | 17.49 (13.99-20.45) | 0.084 |
| CRP (mg/L) | 2.32 (0.50-12.45) | 2.95 (0.50-15.65) | 3.45 (1.35-26.22) | 0.192 | 2.40 (0.50-12.91) | 3.00 (0.5-15.2) | 3.14 (0.71-16.74) | 0.738 |
| Characteristics | rs3087459 [Median (P_25_-P_75_)] | | | P^*^ | rs4145451 [Median (P_25_-P_75_)] | | | P^*^ |
|  | CC | CA | AA |  | CC | CA | AA |  |
| C3 (g/L） | 0.99 (0.54-1.06) | 0.68 (0.42-0.91) | 0.76 (0.52-0.95) | 0.225 | 0.73 (0.46-0.95) | 0.70 (0.42-0.91) | 0.80 (0.55-0.97) | 0.088 |
| C4 (g/L) | 0.23 (0.18-0.27) | 0.12 (0.07-0.20) | 0.16 (0.07-0.23) | 0.126 | 0.15 (00.07-0.23) | 0.13 (0.07-0.22) | 0.16 (0.10-0.27) | 0.072 |
| ESR (mm/H) | 5.00 (3.50-13.15) | 27.00 (11.25-54.00) | 25.00 (11.00-51.00) | 0.767 | 24.5 (11.75-52.75) | 26.00 (10.00-55.00) | 22.00 (12.25-40.75) | 0.8 |
| RF (IU/ml) | –^**^ | 9.82 (8.93-15.15) | 9.70 (8.15-11.03) | 0.405 | 9.60 (8.20-10.95) | 10.10 (9.00-13.63) | 9.90 (7.23-13.68) | 0.713 |
| IgA (mg/L) | 2.97 (2.21-3.77) | 2.53 (1.70-3.20) | 2.54 (1.90-3.42) | 0.402 | 2.77 (2.05-3.92) | 2.50 (1.72-3.30) | 2.56 (1.91-3.04) | 0.651 |
| IgM (mg/L) | 1.34 (0.73-1.87) | 1.02 (0.69-1.26) | 0.98 (0.69-1.50) | 0.5 | 0.91 (0.71-1.40) | 1.10 (0.69-1.46) | 0.87 (0.63-1.50) | 0.433 |
| IgG (g/L) | 14.21 (9.86-21.46) | 15.30 (11.15-20.70) | 13.70 (10.54-18.87) | 0.218 | 14.26 (10.98-21.37) | 14.77 (10.29-20.77) | 13.70 (11.10-17.60) | 0.338 |
| CRP (mg/L) | 4.32 (2.00-23.37) | 2.95 (0.50-15.77) | 2.84 (0.51-13.27) | 0.778 | 2.67 (0.62-17.70) | 3.00 (0.50-12.61) | 3.14 (0.59-35.25) | 0.657 |
| Characteristics | rs6458155 [Median (P_25_-P_75_)] | | | P^*^ | rs9369217 [Median (P_25_-P_75_)] | | | P^*^ |
|  | TT | TC | CC |  | TT | TC | CC |  |
| C3 (g/L） | 0.79 (0.57-0.99) | 0.69 (0.42-0.93) | 0.73 (0.47-0.95) | 0.969 | 0.77 (0.28-1.07) | 0.72 (0.51-0.95) | 0.74 (0.44-0.94) | 0.999 |
| C4 (g/L) | 0.16 (0.11-0.23) | 0.13 (0.07-0.22) | 0.15 (0.07-0.22) | 0.949 | 0.19 (0.08-0.27) | 0.13 (0.07-0.23) | 0.15 (0.07-0.22) | 0.981 |
| ESR (mm/H) | 27.00 (10.50-47.00) | 28.00 (11.00-51.00) | 23.00 (11.00-52.75) | 0.72 | 5.00 (2.75-41.00) | 23.00 (10.00-40.00) | 28.00 (12.5-56.50) | 0.144 |
| RF (IU/ml) | 10.30 (8.40-14.20) | 10.10 (9.00-13.50) | 9.20 (7.80-10.10) | 0.019 | –^**^ | 9.70 (7.45-16.00) | 10.10 (8.50-11.70) | 0.599 |
| IgA (mg/L) | 2.57 (2.12-3.13) | 2.47 (1.74-3.30) | 2.62 (1.74-3.70) | 0.393 | –^**^ | 2.56 (1.66 -3.28) | 2.54 (1.84-3.40) | 0.624 |
| IgM (mg/L) | 1.10 (0.69-1.51) | 1.08 (0.69-1.46) | 0.91 (0.68-1.31) | 0.357 | –^**^ | 13.89 (10.48-20.04) | 14.43 (10.62-19.74) | 0.893 |
| IgG (g/L) | 13.36 (11.15-17.48) | 15.03 (10.51-20.54) | 13.89 (10.25-21.22) | 0.652 | –^**^ | 2.95 (0.56-13.83) | 3.07 (0.61-16.51) | 0.425 |
| CRP (mg/L) | 3.23 (0.69-45.49) | 2.80 (0.50-12.23) | 3.20 (0.62-16.56) | 0.72 | –^**^ | 0.94 (0.65-1.26) | 1.08 (0.70-1.49) | 0.125 |

^*^(wild-type genotypes + heterozygosity) vs mutant-type genotypes. For example, GG+GA versus AA genotype for rs5369.

^**^Less than four positive or negative results for this index.

SLE, systemic lupus erythematosustis; ESR, erythrocyte sedimentation rate; RF, rheumatoid factors; CRP, C-reactive protein.
