# Supplementary material for: Association between endothelin-1 and systemic lupus erythematosus: insights from a case–control study
Source: Sci Rep. 2023 Sep 25;13:15970. doi: 10.1038/s41598-023-43350-0 (PMC10520074; doi:10.1038/s41598-023-43350-0)
Supplement: Supplementary file 11 — Supplementary Table 11. [file 41598_2023_43350_MOESM11_ESM.docx]

Supplementary table 11 Primer information about *ET-1* gene polymorphisms.

| ID | Primer AlleleX | Primer AlleleX | Primer Common | AlleleX | AlleleY |
| --- | --- | --- | --- | --- | --- |
| rs5369 | CCACAAAGGCAACAGACCGTGAA | CACAAAGGCAACAGACCGTGAG | TCTTTTTGGCTAGCACATTGGCATCTATT | A | G |
| rs5370 | ATGATCCCAAGCTGAAAGGCAAG | CATGATCCCAAGCTGAAAGGCAAT | ATGTGCTCGGTTGTGGGTCACATAA | G | T |
| rs1476046 | CAAAGTGTTGAAGCCATTTGTGCAGA | AAAGTGTTGAAGCCATTTGTGCAGG | GCTCCAGTTCTAGCTCTGCTACAAA | A | G |
| rs2070699 | GGAGCCAGCGCTAATGAATGAC | GGAGCCAGCGCTAATGAATGAA | GTCTCTAGAGGGCATTGTAACCCTA | G | T |
| rs2071942 | GTATTTACCACTTTCCCTGAGAAATCA | ATTTACCACTTTCCCTGAGAAATCG | TCTAAATGTCCGCTCCCCAAAATGATTTT | A | G |
| rs2071943 | TTGAATAGTTATGGCCATCTTAATAAATTGAT | GAATAGTTATGGCCATCTTAATAAATTGAC | TGGGCCTACTGTATGCTTCTTTTCTTTT | A | G |
| rs3087459 | CATTCCTCCTTCCTATGTTAGGCAA | CCTCCTTCCTATGTTAGGCAC | GGCAGAAAAAAACGCAACCTGGCTT | A | C |
| rs4145451 | ATTATTTTTGGAAAAATGATTATTCTGTCCT | ATTATTTTTGGAAAAATGATTATTCTGTCCG | GTAGGTATGGAAAAGTGGGTTTCAGATAA | A | C |
| rs6458155 | CCTGATGACGTAATTCCCCTCG | CCCTGATGACGTAATTCCCCTCA | GGAGATGAGTAGATGCACGTTGAGAA | C | T |
| rs9369217 | CATTCATGGTGTGGACTGTGTCG | CATTCATGGTGTGGACTGTGTCA | GTGGAGTTGGAGATTTGAACTCATACAAT | C | T |
